# Farnesoid x Receptor Deficiency Promotes Hepatocytic Injury in *Cyp2c70*‐Deficient Mice With a Human‐Like Bile Acid Composition

**DOI:** 10.1111/liv.70632

**Published:** 2026-04-09

**Authors:** Hilde D. de Vries, Jinxiao Li, Kirill Ustyantsev, Milaine V. Hovingh, Niels L. Mulder, Rick Havinga, Anna Palmiotti, Krisztina de Bruyn, Brecht Attema, Ellen Weersing, Rachel E. Thomas, Vincent W. Bloks, Eugene Berezikov, Martijn van Faassen, Henkjan J. Verkade, Jan Freark de Boer, Folkert Kuipers

**Affiliations:** ^1^ Department of Pediatrics University of Groningen, University Medical Center Groningen (UMCG) Groningen the Netherlands; ^2^ European Research Institute for the Biology of Ageing (ERIBA), University of Groningen University Medical Center Groningen (UMCG) Groningen the Netherlands; ^3^ Department of Laboratory Medicine, University of Groningen University Medical Center Groningen (UMCG) Groningen the Netherlands; ^4^ Royal GD Deventer the Netherlands

**Keywords:** bile acids, humanized mouse model, liver, nuclear receptor, progressive familial intrahepatic cholestasis

## Abstract

**Background and Aims:**

Loss‐of‐function mutations in bile acid (BA)‐activated farnesoid x receptor (*FXR*/*NR1H4*) cause severe neonatal liver pathology in humans, earlier referred to as progressive familial intrahepatic cholestasis type 5 (PFIC5). However, *Fxr*‐deficient mice do not develop early‐onset liver disease, possibly due to the predominance of hydrophilic muricholic acids (MCAs) in their BA pool. Mice lacking *Cyp2c70* display a human‐like BA composition, lacking MCAs. This study aimed to evaluate whether *Fxr/Cyp2c70‐*double knockout (DKO) mice recapitulate the pathophysiology of human FXR‐deficiency.

**Methods:**

BA metabolism and liver pathology were assessed in wild‐type (WT), *Fxr*‐knockout (KO), *Cyp2c70*‐KO and DKO mice of both sexes.

**Results:**

*Fxr*‐deficiency markedly reduced the amounts of bile salt export pump (BSEP/ABCB11), but did not affect biliary BA secretion. DKO mice showed exacerbated hepatocytic injury and inflammation compared to *Fxr*‐KO mice of both sexes. Surprisingly, despite higher alanine aminotransferase levels that indicate hepatocellular damage, female DKO mice showed much less liver fibrosis and ductular reactions than female *Cyp2c70*‐KO mice. Gene Set Enrichment Analysis suggested that epithelial–mesenchymal transition was upregulated in *Cyp2c70*‐KO livers compared to WT mice but downregulated particularly in livers of female DKO mice compared to *Cyp2c70*‐KO. Biliary BA hydrophobicity was increased by the deletion of *Cyp2c70*, yet reduced by simultaneous absence of *Fxr* in female mice.

**Conclusions:**

*Fxr/Cyp2c70*‐DKO mice exhibit more severe hepatocytic injury than *Fxr*‐KO mice, mirroring the clinical phenotype of FXR deficiency. These data highlight the importance of studying the aetiology of cholestatic liver diseases in the context of a human‐like BA composition.

## Summary

- Loss‐of‐function mutations in bile acid (BA)‐activated farnesoid x receptor (FXR) in humans cause severe neonatal liver pathology, whereas *Fxr*‐deficient mice do not develop early‐onset liver disease, possibly due to the predominance of hydrophilic muricholic acids (MCAs) in their BA pool, produced by CYP2C70.
- In this study, we generated *Fxr/Cyp2c70*‐double knockout (DKO) mice to evaluate whether DKO mice in the context of a human‐like BA composition recapitulate the pathophysiology of human FXR‐deficiency.
- *Fxr/Cyp2c70*‐DKO mice exhibit more severe hepatocytic injury and inflammation than *Fxr*‐KO mice, mirroring the clinical phenotype of FXR‐deficiency and highlighting the importance of studying the aetiology of cholestatic liver diseases in the context of a human‐like BA composition.

**Abbreviations**
ALT: alanine aminotransferase
AST: aspartate aminotransferase
BA: bile acid
BSEP: bile salt export pump
BW: body weight
CA: cholic acid
C4: 7α‐hydroxy‐4‐cholesten‐3‐one
CAR: constitutive androstane receptor
CDCA: chenodeoxycholic acid
DKO: double knockout
EMT: epithelial–mesenchymal transition
FDR: false discovery rate
FISH: fluorescence in situ hybridization
FSP1: fibroblast‐specific protein 1
FXR: farnesoid x receptor
GSEA: gene set enrichment analysis
H&E: haematoxylin and eosin
Het: heterozygous
HI: hydrophobicity index
IL‐1β: interleukin 1β
KO: knockout
LW: liver weight
MCAs: muricholic acids
NTCP: Na^+ ^taurocholate co‐transporting polypeptide
PC: principal component
PCA: principal component analysis
PFIC5: progressive familial intrahepatic cholestasis type 5
PXR: pregnane X receptor
SRFG: sirius red/fast green
THBAs: tetrahydroxylated bile acids
WT: wild‐type

## 1 Introduction

Farnesoid x receptor (FXR/*NR1H4*) is a bile acid (BA)‐activated nuclear receptor [1] that functions as the regulator of hepatic BA synthesis. FXR also regulates BA transport, highlighting its critical role in the maintenance of BA homeostasis. Activation of FXR reduces expression of Na^+ ^taurocholate co‐transporting polypeptide (NTCP/*SLC10A1*) and increases expression of the bile salt export pump (BSEP/*ABCB11*), mediating hepatic BA import and their canalicular export, respectively [2]. By inhibiting hepatic BA synthesis and import, FXR prevents accumulation of BAs within hepatocytes. In line with its actions as an orchestrator of BA homeostasis, loss‐of‐function mutations in human FXR have been shown to underly a specific hereditary cholestatic liver disease that is a member of the expanding family of progressive familial intrahepatic cholestasis (PFIC) types [3].

PFIC is an umbrella term used to describe a heterogenous group of diseases caused by mutations in genes involved in bile formation. Regardless of the genetic cause, PFIC is associated with impaired bile formation with the first symptoms appearing in early life and progression to severe liver disease during childhood [4]. In 2016, Gomez‐Ospina and colleagues [3] reported four patients from two different families with homozygous mutations in *NR1H4* and severe neonatal cholestasis, which at that time was classified as PFIC5. According to histological analyses of the livers of these patients [3, 5], BSEP expression was absent and elevated plasma BA and bilirubin levels indicated the presence of severe cholestasis. More recently, 12 other patients with pathological mutations in *NR1H4* have been described, all presenting with severe liver disease at an early age [5, 6, 7, 8, 9, 10, 11, 12]. Absence of functional FXR leads to high plasma levels of 7α‐hydroxy‐4‐cholesten‐3‐one (C4) [3], a biomarker of hepatic BA synthesis, likely indicating augmented activity of the rate controlling BA synthesis enzyme cytochrome P450 family 7 subfamily A member 1 (CYP7A1) and, consequently, an increased BA synthesis rate. Due to the current lack of treatment options, all FXR‐deficient patients that have been described so far required liver transplantation and/or died within the first year of life [3, 5, 6, 7, 8, 9, 10, 11, 12].

Mouse models have been extensively used to study mechanisms underlying cholestatic liver diseases. However, when the genetic defects causing the various PFIC subtypes were introduced in mice, their phenotypes generally did not accurately reflect the human disease pathology [13, 14, 15, 16]. Indeed, *Fxr*‐deficiency in mice does, in sharp contrast to humans, not cause severe (histological) liver abnormalities or cholestasis in early life [17, 18]. We hypothesized that these differences are, at least in part, driven by the abundant presence of specific hydrophilic BAs, that is, muricholic acids (MCAs), in mice. High concentrations of hydrophobic BAs, such as chenodeoxycholic acid (CDCA), which are abundantly present in the human BA pool, are toxic and can induce liver damage [19]. In contrast, hydrophilic MCAs are not toxic and are even reported to have cytoprotective properties [20], which might explain the absence of severe liver disease in mice with *Fxr*‐deficiency.

We have generated *Cyp2c70*‐deficient mice that lack the MCA‐synthesizing enzyme CYP2C70 and, accordingly, display a human‐like BA composition with substantial amounts of CDCA [21]. We observed previously that female *Cyp2c70*‐deficient mice, but not the males, develop cholanemia and liver fibrosis during ageing [21]. In addition, we have previously characterized BA homeostasis in *Fxr*‐deficient wildtype (WT) mice, showing an enlarged BA pool size containing MCAs and an increased hepatic BA synthesis rate [18]. In the current study, we cross‐bred *Cyp2c70*‐deficient mice with *Fxr*‐deficient mice to generate *Fxr/Cyp2c70*‐double knockout (DKO) mice and evaluated their phenotype in males and females in order to delineate the role of FXR on BA‐induced liver pathology in the context of a human‐like BA composition and, thereby, to evaluate whether DKO mice recapitulate the pathophysiology of human FXR‐deficiency.

## 2 Methods

### 2.1 Animals

*Fxr*‐KO mice and *Cyp2c70‐*KO mice on a C57BL/6J background were previously described by our group [18, 21]. To generate DKO animals, female *Fxr*‐KO mice were cross‐bred with male *Cyp2c70*‐KO. Both male and female WT, *Fxr‐*KO, *Cyp2c70*‐KO and DKO mice were used in the experiment. Mice were terminated following bile cannulation at the age of 12–15 weeks. Additional experiment details are described in the Supplemental Methods. All animal procedures complied with Dutch law and were approved by the Dutch Central Committee for Animal Experiments and the responsible Animal Welfare Body of the University of Groningen.

### 2.2 Quantitative RT‐PCR

RNA isolation, complementary DNA synthesis, and quantitative RT‐PCR were performed as described in the Supplemental Methods. Relative gene expression was normalized to *Cyclophilin G*, with WT male mice as the reference group.

### 2.3 Measurements of Bile Acids and Plasma C4

BA levels in plasma and bile were measured by using (U)HPLC‐MS/MS on a Xevo TQ‐XS Triple Quadrupole Mass Spectrometer coupled to an Acquity UPLC system (Waters, Milford, MA) and quantified using stable isotopically labelled internal standard. Plasma C4 was measured using a Xevo TQ‐S micro Triple Quadrupole Mass Spectrometer (Waters, Milford, MA) and quantified using deuterium labelled C4 (Toronto Research Chemicals, Toronto, Canada).

### 2.4 Western Blotting

Protein abundance was analysed as described in the Supplemental Methods. Band intensities were quantified using Image Lab (v5.2.1, Bio‐Rad Laboratories, California, USA). Target protein intensities were normalized to those of HSP90 as a loading control.

### 2.5 Histological Analysis

Liver tissues were fixed in 4% (w/v) formalin, embedded in paraffin, and sectioned at 4 μm. Haematoxylin & eosin (H&E) and sirius red/fast green (SRFG) staining were performed using standard protocols. Images were acquired using a Hamamatsu NanoZoomer (Hamamatsu Photonics, Almere, the Netherlands). Liver H&E‐stained sections were histologically assessed for hepatocellular single‐cell death, pigmented macrophages, hepatocellular ballooning and steatosis with semi‐quantitative scoring performed by a board‐certified veterinary pathologist in a blinded manner. Additional details regarding histological scoring criteria, immunofluorescent and immunohisto chemistry staining, and OneSABER TSA fluorescence in situ hybridization (FISH) [22] staining are provided in the Supplemental Methods. Stained areas were quantified using ImageJ (v1.54d, National Institutes of Health, Bethesda, MD).

### 2.6 Plasma Parameter Measurements

Plasma alanine aminotransferase (ALT), aspartate aminotransferase (AST), and bilirubin were determined using a Cobas 6000 analyzer with standard reagents (Roche Diagnostics, the Netherlands).

### 2.7 Blood Cell Counts

Peripheral blood was collected from the retro‐orbital plexus in heparinized capillary tubes. Cell counts were determined using 25 μL of blood using Smart V5 (A. Menarini Diagnostics, Belgium).

### 2.8 RNA Sequencing

RNA library preparation and sequencing strategy are described in the Supplemental Methods. Gene counts were normalized, and differential gene expression was determined using DESeq2 (v1.46.0) with false discovery rate (FDR) correction applied via Benjamini‐Hochberg method. Gene Set Enrichment Analysis (GSEA) was conducted using clusterProfiler (v4.14.6) to identify enriched pathways.

### 2.9 Statistical Analysis

Data are presented as bar plots or Tukey box‐and‐whisker plots using GraphPad Prism (v9.0, Boston, Massachusetts, USA). Mendelian ratios were analysed using Pearson's Chi‐square test, while contingencies were calculated using Fisher's exact test (GraphPad Prism). Statistical significance of nonparametric comparisons was assessed using the Kruskal‐Wallis H test with Conover post hoc comparisons (Brightstat online tool [23]). *P* < 0.05 were considered statistically significant.

## 3 Results

### 3.1 *Fxr*‐Deficiency in the Context of a Human‐Like Bile Acid Composition Does Not Affect Birth Rate and Growth but Does Impact Bile Acid Homeostasis

**FIGURE 1:**
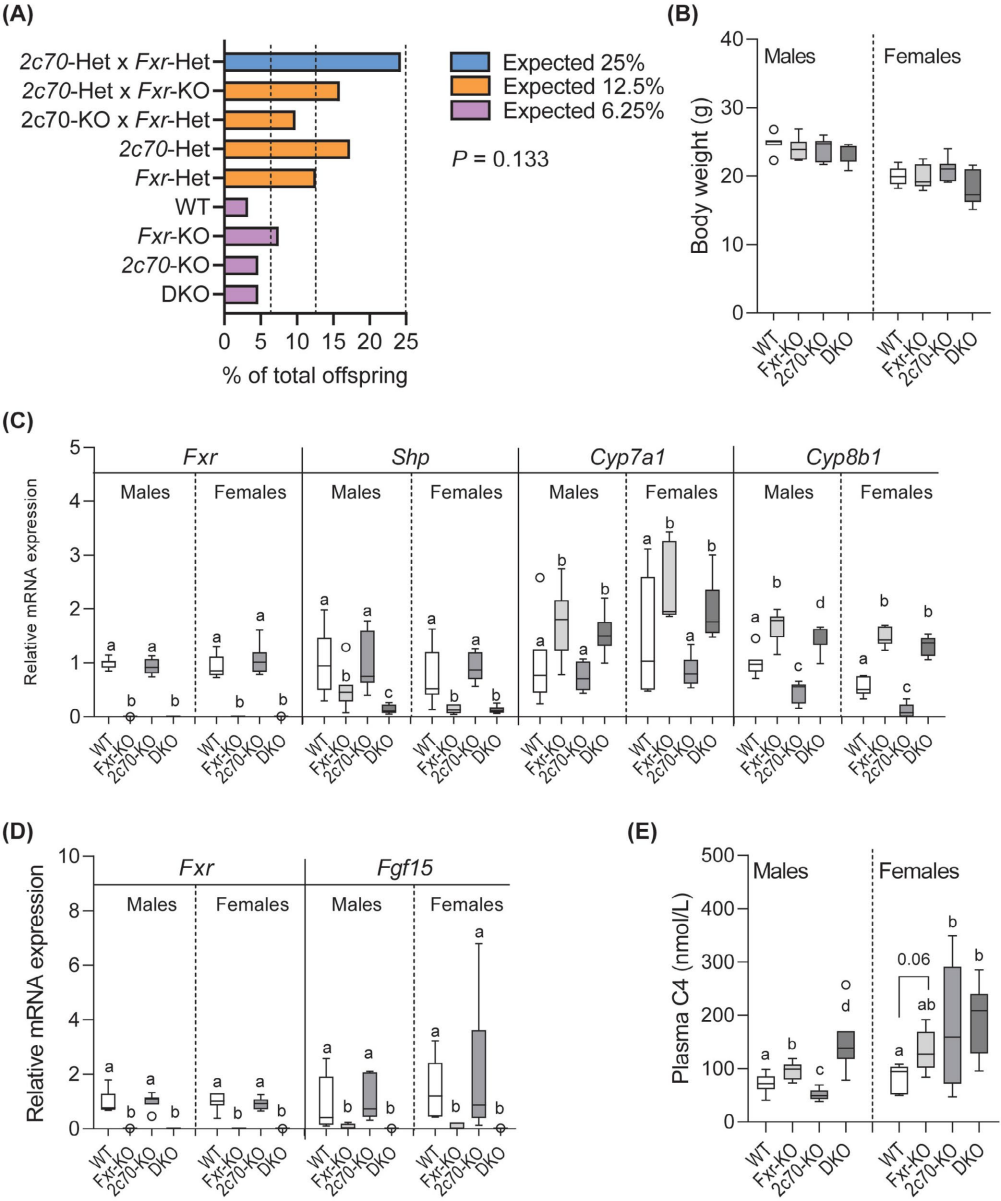
*Fxr*‐deficiency in the context of a human‐like bile acid composition does not affect birth rate and growth but dysregulates bile acid synthesis. (A) Genotype distribution of offspring from *Cyp2c70*‐Het/*Fxr*‐Het breeding pairs (*n* = 214). Mendelian ratios were tested using Pearson's Chi‐square test. (B) Body weight of WT, *Fxr*‐KO, *2c70*‐KO and DKO mice at 9–16 weeks of age (*n* = 6–9 mice/group). (C) Hepatic mRNA expression of *Fxr*, *Shp*, *Cyp7a1*, and *Cyp8b1* (*n* = 6–9 mice/group). (D) Intestinal mRNA expression of *Fxr* and *Fgf15* (*n* = 6–9 mice/group). (E) Plasma C4 levels, a marker of BA synthesis (*n* = 6–9 mice/group). Data are presented as bar plot or Tukey box‐and‐whisker plots. Groups with the same letters or without letters are not significantly different, whereas groups with different letters differ significantly (*p* < 0.05), assessed using the Kruskal‐Wallis H test with Conover post hoc comparisons. WT, wild type; Het, heterozygous; KO, knockout; DKO, double knockout; *2c70*, *Cyp2c70*; C4, 7α‐hydroxy‐4‐cholesten‐3‐one; BA, bile acid.

Given that homozygous *FXR* mutations in humans are associated with severe liver disease and fatal complications during the neonatal period [3], we first monitored survival rates of DKO mice. At weaning (4 weeks of age), the observed genotype distribution did not differ from the expected Mendelian ratios (Chi‐square test, *p* = 0.133) (Figure 1A), indicating that DKO mice do not experience a survival disadvantage in utero or during the suckling period. In adulthood, DKO mice showed no significant differences in body weight or overall appearance compared to WT, *Fxr*‐KO, or *Cyp2c70*‐KO mice (Figure 1B), indicating *Fxr*‐deficiency does not impair normal growth and development.

Next, we compared the impact of *Fxr* deletion in the context of the murine or human BA composition on the expression of its downstream genes. Transcripts of *Fxr* were absent in *Fxr*‐KO and DKO mice in both liver and intestine (Figure 1C,D), confirming effective KO. In liver, *Shp* expression was decreased, while expression of BA synthesis genes *Cyp7a1* and *Cyp8b1* was increased in *Fxr*‐KO and DKO mice (Figure 1C). In the intestine, absence of *Fxr* led to markedly decreased *Fgf15* expression irrespective of the *Cyp2c70* genotype (Figure 1D), demonstrating suppression of the *Fxr‐Fgf15* signalling axis. Consistently, plasma C4 levels were elevated in male *Fxr*‐KO and DKO mice, with DKO mice showing significantly higher levels than *Fxr*‐KO mice (Figure 1E). In females, *Fxr*‐KO mice showed a tendency towards increased C4 levels (*p* = 0.06), while DKO females did not exhibit significant changes compared to *Cyp2c70*‐KO mice.

**FIGURE 2:**
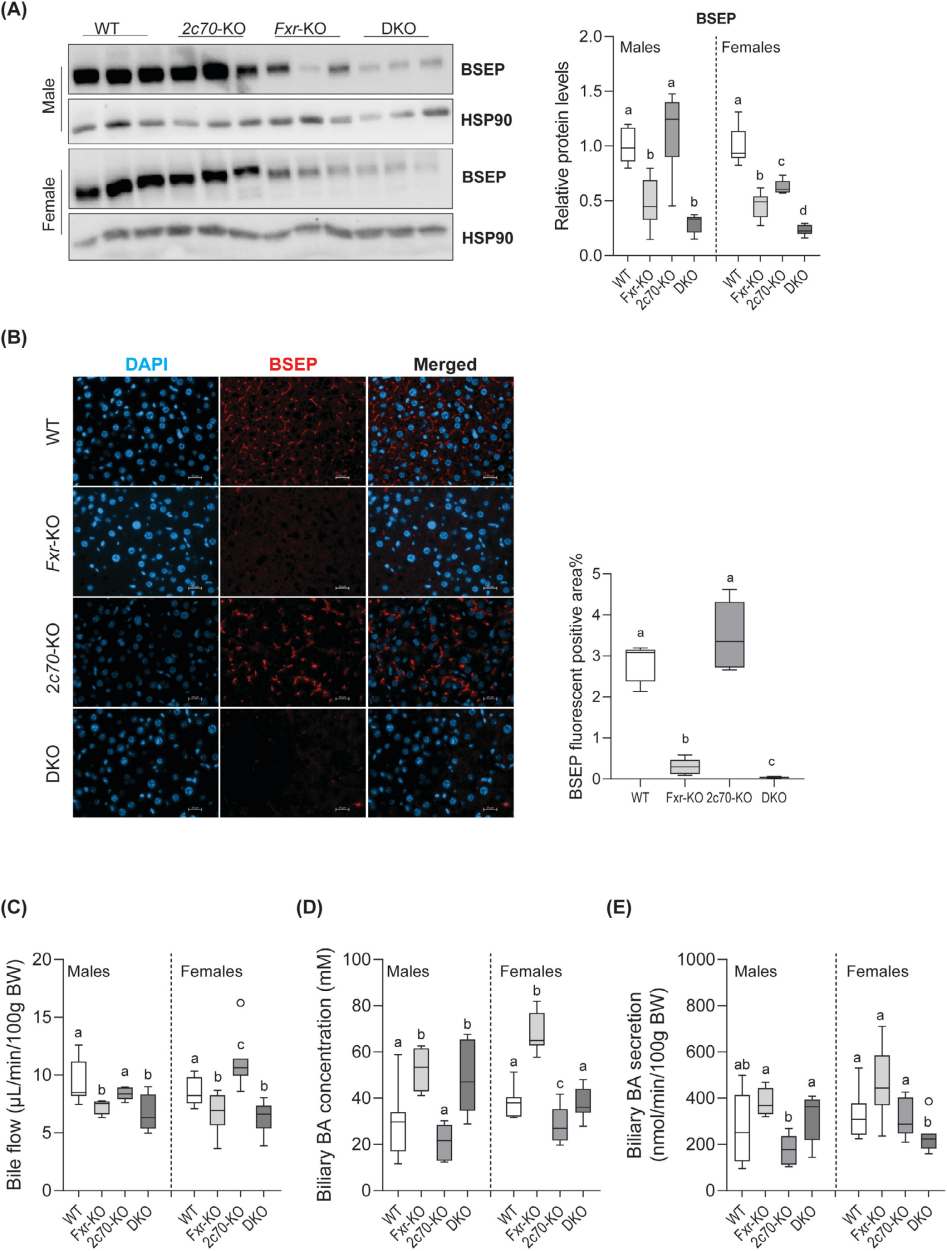
*Fxr*‐deficiency reduces BSEP abundance at the canalicular membrane and impairs bile flow. (A) Immunoblotting (left) liver lysates for BSEP and HSP90 (loading control). Quantified band intensities were normalized to HSP90 protein (*n* = 5–7 mice/group; right). (B) Representative immunofluorescence images (left) showing BSEP localization in liver sections from female mice. Quantified BSEP fluorescent positive area (%; right). (C) Bile flow (*n* = 5–7 mice/group). (D) Biliary BA concentrations (*n* = 5–7 mice/group). (E) Biliary BA secretion rates, calculated by multiplying bile flow with biliary BA concentrations (*n* = 5–7 mice/group). Data are presented as Tukey box‐and‐whisker plots. Groups with the same letters are not significantly different, whereas groups with different letters differ significantly (*p* < 0.05), assessed using the Kruskal‐Wallis H test with Conover post hoc comparisons. BA, bile acid; WT, wild type; Het, heterozygous; KO, knockout; DKO, double knockout; *2c70*, *Cyp2c70*.

As FXR regulates BSEP, we assessed hepatic expression of this transporter mediating hepatobiliary BA secretion. *Bsep* mRNA was reduced in both *Fxr*‐KO and DKO mice compared to WT and *Cyp2c70*‐KO mice, with DKO animals showing significantly lower expression levels than *Fxr*‐KO (Figure S1A). Consistent with these findings, BSEP protein levels were markedly decreased in *Fxr*‐KO and DKO livers in both males and females, with DKO showing barely detectable BSEP protein (Figure 2A,B). Bile cannulation revealed that bile flow was reduced in *Fxr*‐KO and DKO mice, without significant differences between these two groups (Figure 2C). The reduction in bile flow in these mice was, however, associated with higher biliary BA concentrations (Figure 2D). Consequently, biliary BA secretion rates were only minorly decreased in DKO females compared to the other groups (Figure 2E) despite low presence of BSEP in the livers of these *Fxr*‐KO and DKO mice.

### 3.2 DKO Mice Exhibit Exacerbated Hepatic Injury

**FIGURE 3:**
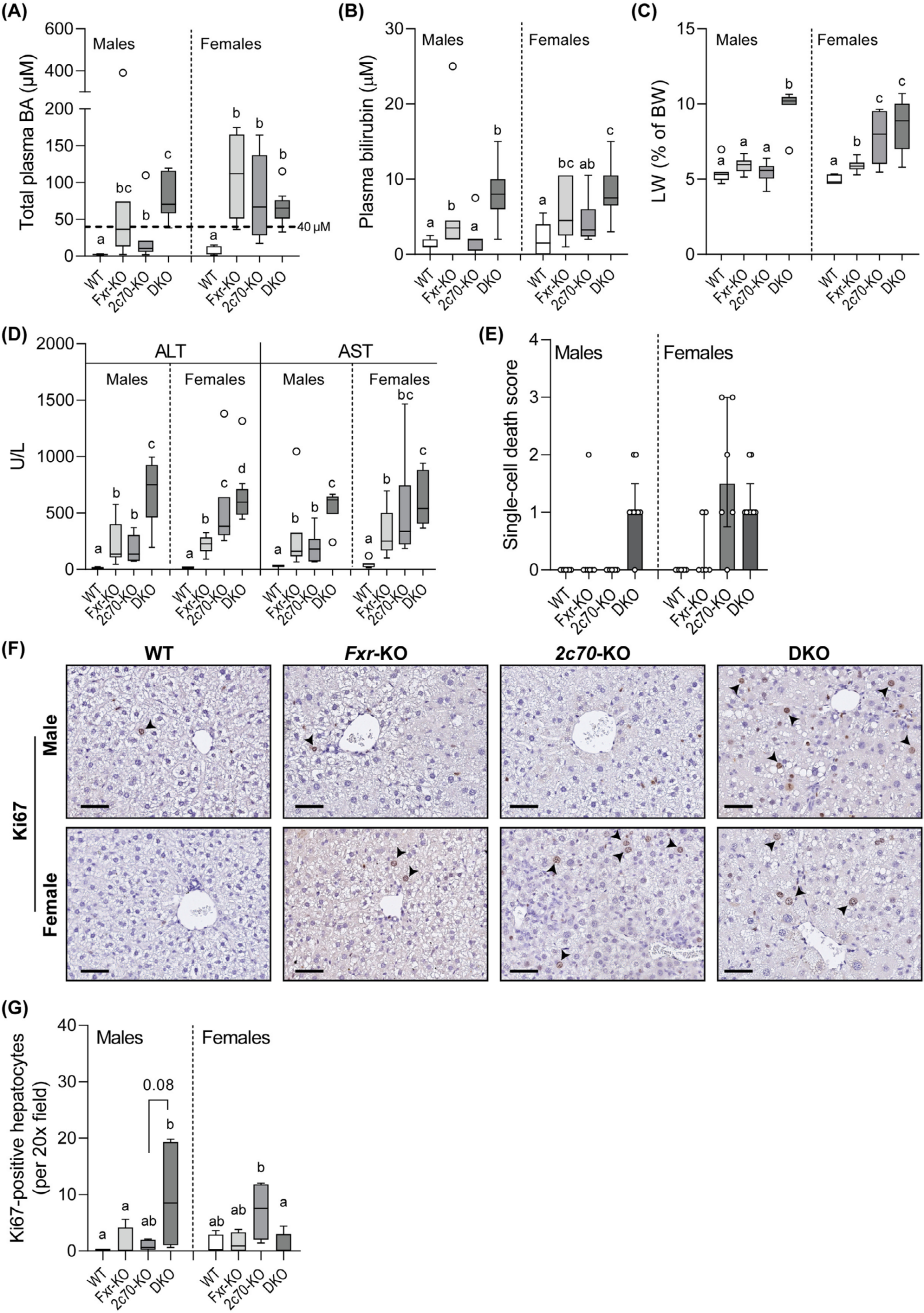
DKO mice develop hypercholanemia and exhibit hepatic injury. (A) Total plasma BA concentrations and (B) plasma bilirubin concentrations (*n* = 6–9 mice/group). (C) Liver weight as percentage of body weight (*n* = 6–9 mice/group). (D) Plasma concentrations of ALT and AST (*n* = 6–9 mice/group). (E) Hepatocyte single‐cell death score, assessed by a veterinary pathologist on H&E‐stained liver sections (*n* = 6–9 mice/group). (F) Representative liver sections stained for Ki‐67 positive hepatocytes (brown nuclei, arrowheads). Scale bars: 50 μm. (G) Quantification of Ki‐67 positive hepatocytes. At least five randomly selected fields per liver were counted. Mean values of positive cell numbers per mouse were calculated from these fields to represent individual mice (*n* = 4–5 mice/group). Data are presented as Tukey box‐and‐whisker plots or a bar plot showing median with interquartile range. Groups with the same letters are not significantly different, whereas groups with different letters differ significantly (*p* < 0.05), assessed using the Kruskal‐Wallis H test with Conover post hoc comparisons. BA, bile acid; WT, wild type; Het, heterozygous; KO, knockout; DKO, double knockout; *2c70*, *Cyp2c70*; LW, liver weight; BW, body weight.

We next evaluated the consequences of *Fxr*‐deficiency in the context of a human‐like BA composition for liver function. Plasma BA levels were significantly elevated in all knockout mice compared to WT mice (Figure 3A). In males, 86% (6/7) of DKO mice exhibited hypercholanemia (plasma BA levels ≥ 40 μM) [24], which was a higher percentage than *Cyp2c70*‐KO (14%, 1/7, *p* < 0.05). In contrast, the prevalence of hypercholanemia was much less pronounced when *Fxr* was depleted in the context of a normal murine BA composition (2/7 vs. 0/7, *p* = 0.46, for *Fxr*‐KO vs. WT, respectively). In females, the prevalence of hypercholanemia in DKO mice (89%) was comparable to those observed in *Fxr*‐KO (83%) and *Cyp2c70*‐KO (67%). In both *Fxr*‐KO and DKO mice, cholic acid (CA) was the dominant plasma BA, whereas CDCA was the most abundant BA in *Cyp2c70*‐KO mice (Figure S2). Plasma bilirubin levels were significantly elevated in *Fxr*‐KO compared to WT mice and showed a tendency of further elevation in male (*p* = 0.09) and female (*p* = 0.08) DKO mice compared to *Fxr*‐KO mice (Figure 3B).

Notably, male DKO mice exhibited a two‐fold increase in liver‐to‐body weight ratio compared to all other groups. Female DKO mice exhibited higher liver‐to‐body weight ratios than WT and *Fxr*‐KO mice as well, but liver weights did not increase further when *Fxr* was knocked out on a background of *Cyp2c70‐*deficiency (Figure 3C). Plasma transaminases were significantly elevated in *Fxr*‐KO and *Cyp2c70*‐KO mice compared to WT, but the highest levels in both male and female mice were observed in DKO animals (Figure 3D). This indicates that the latter group displayed the most extensive hepatocyte damage. Consistent with this, hepatocellular single‐cell death scores were markedly increased in male DKO livers as well as in female *Cyp2c70*‐KO and DKO livers (Figure 3E).

To determine whether this damage induced compensatory proliferation, Ki‐67 was analysed in livers. In male mice, Ki‐67 mRNA expression was higher in DKO mice than in all other genotypes (Figure S1B). This was confirmed by immunohistological staining of liver sections, which showed increased numbers of Ki‐67 positive hepatocytes, albeit with more variation (Figure 3F,G). In female groups, *Cyp2c70*‐KO mice showed most hepatocyte proliferation, while inactivation of *Fxr* on the background of *Cyp2c70*‐deficiency was associated with an unforeseen reduction of hepatocyte proliferation in females (Figure 3F,G), suggesting sex‐dependent effects of *Fxr*‐deficiency on hepatocyte cell division in this model.

### 3.3 Inflammatory Responses Are Particularly Pronounced in Male DKO Mice

**FIGURE 4:**
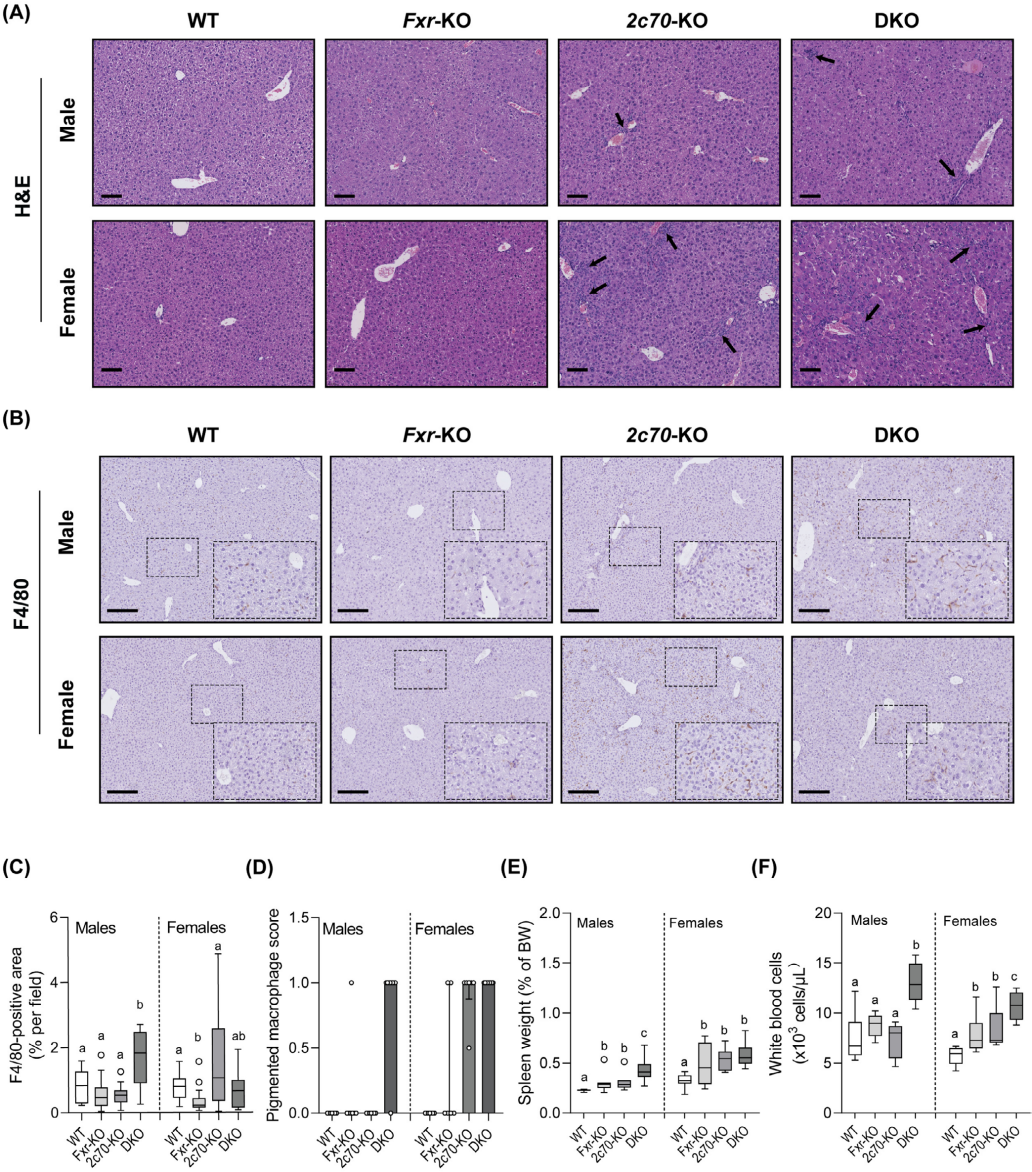
DKO mice, especially in males, exhibit increased immune responses. (A) Representative H&E staining of liver sections. The black arrows point to immune cell infiltration regions. Scale bars: 100 μm. (B) Representative liver sections stained for F4/80 (*n* = 4–5 mice/group). Scale bars: 200 μm. (C) Quantification of F4/80 positive‐stained area as the percentage of total field area. (D) Pigmented macrophage score, assessed by a veterinary pathologist on H&E‐stained liver sections (*n* = 6–9 mice/group). (E) Spleen weight as percentage of body weight (*n* = 6–9 mice/group). (F) Circulating white blood cell numbers (*n* = 5–8 mice/group). Data are presented as Tukey box‐and‐whisker plots or a bar plot showing the median with interquartile range. Groups with the same letters are not significantly different, whereas groups with different letters differ significantly (*p* < 0.05), assessed using the Kruskal‐Wallis H test with Conover post hoc comparisons. H&E, Haematoxylin and Eosin; WT, wild type; Het, heterozygous; KO, knockout; DKO, double knockout; *2c70*, *Cyp2c70*; BW, body weight.

More detailed histological examination revealed periportal immune cell infiltration in liver sections from male and female *Cyp2c70*‐KO and DKO mice (Figure 4A). In addition, mRNA expression of the macrophage marker *F4/80* (*Adgre1*, Figure S1C) and amounts of F4/80+ cells were increased in livers of DKO males compared to other genotypes (Figure 4B,C), whereas females showed considerable variation in hepatic macrophage content. Semi‐quantification of pigmented macrophages confirmed the highest macrophage scores in male DKO mice, while in females elevated scores were observed in both *Cyp2c70*‐KO and DKO mice (Figure 4D). In line with these findings, the mRNA expression of genes encoding inflammatory factors, such as the chemokine *Ccl2* (*Mcp‐1*), was elevated in livers of male DKO mice (Figure S3), while similar trends were observed in females. Spleen weights were significantly increased in *Fxr*‐KO, *Cyp2c70*‐KO, and DKO mice compared to WT in mice of both sexes. Spleens of male DKO mice were notably larger than those of *Fxr*‐KO and *Cyp2c70*‐KO mice, while more within‐group variation was observed in females (Figure 4E). Circulating white blood cell numbers, an indicator of systemic inflammation, were significantly elevated in female but not in male *Fxr*‐KO and *Cyp2c70*‐KO mice compared to WT (Figure 4F). However, in both sexes, white blood cell counts in DKO mice were significantly higher than in all other genotypes, indicating that *Fxr*‐deficiency in the context of a human‐like BA composition causes substantial systemic inflammation.

### 3.4 In the Context of a Human‐Like Bile Acid Composition, *Fxr*‐Deficiency Confers Protection From Liver Fibrosis and Ductular Reactions in Mice

**FIGURE 5:**
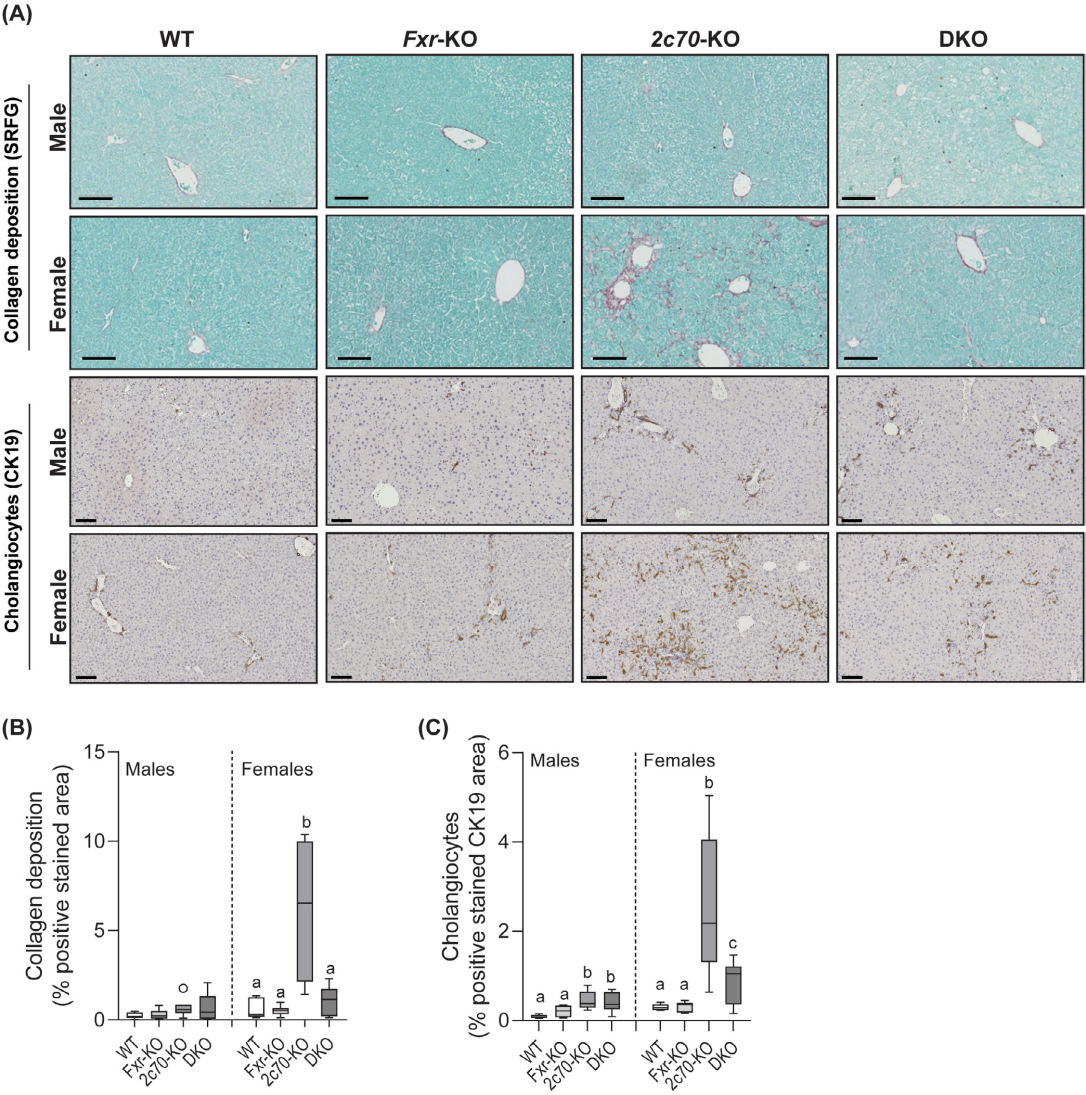
*Fxr*‐deficiency in the context of a human‐like bile acid composition ameliorates liver fibrosis and ductular reactions in female mice. (A) Representative liver sections stained for Sirius Red/Fast Green (collagen deposition) and anti‐CK19 (cholangiocytes). Scale bars: 100 μm. (B) Quantification of collagen deposition and (C) CK19+ cholangiocytes in the liver. Data are presented as Tukey box‐and‐whisker plots. Groups with the same letters or without letters are not significantly different, whereas groups with different letters differ significantly (*p* < 0.05), assessed using the Kruskal‐Wallis H test with Conover post hoc comparisons. WT, wild type; Het, heterozygous; KO, knockout; DKO, double knockout; *2c70*, *Cyp2c70*.

Because the human‐like BA profile in *Cyp2c70*‐KO mice is associated with liver fibrosis and ductular reactions in female mice [21], we next assessed the impact of *Fxr*‐deficiency on these processes. Little fibrosis was observed in the male groups, whereas, in line with the aforementioned observations, *Cyp2c70*‐deficiency was associated with considerable collagen deposition in females. Remarkably, female DKO mice displayed much less collagen deposition than *Cyp2c70*‐KO mice (Figure 5A,B). Moreover, collagen deposition of *Fxr*‐KO and DKO mice was comparable to WT. Ductular reactions were increased in livers of *Cyp2c70*‐KO mice and DKO mice compared to WT and *Fxr*‐KO mice in both males and females. However, CK19+ cholangiocytes were markedly reduced in livers of female DKO mice compared to *Cyp2c70*‐KO mice (Figure 5A,C). Collectively, the data described above indicate that depletion of *Fxr* in the context of a human‐like BA composition, that is, in *Cyp2c70*‐KO mice, aggravates hepatocyte damage but ameliorates ductular reactions and fibrosis.

### 3.5 Gene Set Enrichtment Analysis Indicates That *Fxr*‐Deficiency Reduces Epithelial‐Mesenchymal Transition, Which Is Prominently Upregulated in Female *Cyp2c70*‐KO Mice

**FIGURE 6:**
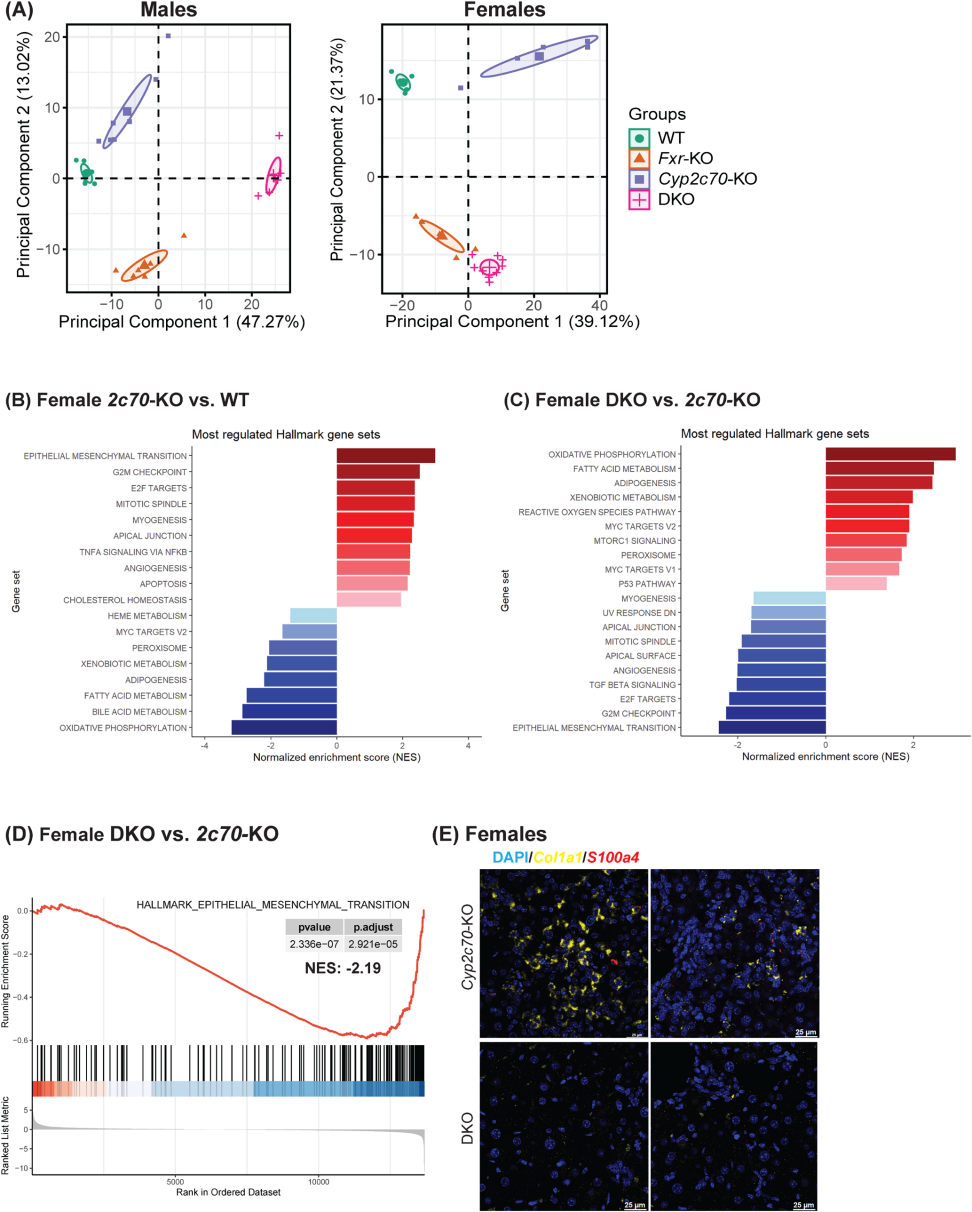
Distinct transcriptome profiles in DKO mice show pronounced downregulation of the Hallmark epithelial–mesenchymal transition gene set. (A) Principal component analysis (PCA) showing the relationship between individual RNA‐seq profiles in males (left) and females (right) (*n* = 6–9 mice/group). Each dot represents a biological repeat, coloured by genotype groups. Distinction is displayed spatially. Similar profiles are clustered close together, while different profiles are separated from each other. (B) Top upregulated and downregulated Hallmark gene sets identified by GSEA in female *2c70*‐KO mice versus WT controls, ranked by stat values (FDR‐adjusted *P* < 0.05). (C) Top upregulated and downregulated Hallmark gene sets identified by GSEA in female DKO mice versus *2c70*‐KO mice, ranked by stat values (FDR‐adjusted *P* < 0.05). (D) GSEA plot of the Hallmark gene set ‘Epithelial–mesenchymal transition’ between female DKO mice versus *2c70*‐KO mice. (E) OneSABER TSA RNA fluorescence in situ hybridization for *Col1a1* (yellow) and *S100a4* (red) in liver sections (DAPI, blue). Random fields from two mice per group. WT, wild type; Het, heterozygous; KO, knockout; DKO, double knockout; *2c70*, *Cyp2c70*.

To shed light on the biological processes contributing to the observed phenotypic manifestations, RNA sequencing was performed on liver samples from all groups. Principal component analysis (PCA) indicated that mice of all genotypes displayed a highly distinct transcriptomic profiles in both male and female animals. Mice separated based on the presence of *Cyp2c70* mainly on principal component (PC) 1 and predominantly on PC2 based on the presence of *Fxr* (Figure 6A). Several major urinary protein (*Mup*) genes were markedly downregulated in both *Cyp2c70*‐KO and DKO livers compared to *Fxr*‐KO and WT controls (Figure S4), consistent with previous findings reporting reduced hepatic *Mup* expression in multiple mouse models of liver disease [25, 26]. Humans, however, do not express *Mup* orthologues. We next performed GSEA using murine Hallmark gene sets [27]. These analyses revealed that epithelial‐mesenchymal transition (EMT) was the most upregulated gene set in female *Cyp2c70*‐KO mice as compared to WT controls (Figure 6B). Remarkably, this gene set was the most downregulated gene set in female DKO compared to *Cyp2c70*‐KO mice (NES = −2.19, *p* < 0.0001; Figure 6C,D, S5). Notably, EMT was the most upregulated Hallmark gene set in male *Cyp2c7*0‐KO vs. WT controls, yet it was not significantly altered in male DKO vs. *Cyp2c70*‐KO mice (Figure S6), suggesting sex‐specific regulation of this process. Furthermore, several cell proliferation‐related Hallmark gene sets that were markedly upregulated in livers from *Cyp2c70*‐KO mice in comparison to WT controls, for example, G2M checkpoint, E2F target, mitotic spindle, were downregulated in the absence of *Fxr* only in females (Figure 6B,C).

Several studies have suggested that hepatic epithelial cells, including hepatocytes and cholangiocytes, can undergo EMT, acquire a fibroblast‐like phenotype and contribute to fibrogenesis [28, 29, 30]. S100A4 (also known as fibroblast‐specific protein 1, FSP1) is a marker to identify EMT‐derived cells in liver injury [28, 29, 30, 31]. To explore whether EMT may be involved in the different degrees of fibrosis between female *Cyp2c70*‐KO and DKO mice, we applied OneSABER TSA FISH^22^ to detect *S100a4* and *Col1a1* mRNA in liver sections. While *Col1a1*‐ and *S100a4*‐positive cells were evident in *Cyp2c70*‐KO liver sections, cells expressing these transcripts were substantially reduced in DKO mice (Figure 6E). Interestingly, *Col1a1*/*S100a4*‐double positive cells were not observed, suggesting that EMT‐derived cells are not a primary source of collagen type I in livers of *Cyp2c70*‐KO mice.

### 3.6 DKO Mice Increase 12α‐Hydroxylation in Bile Acids

**FIGURE 7:**
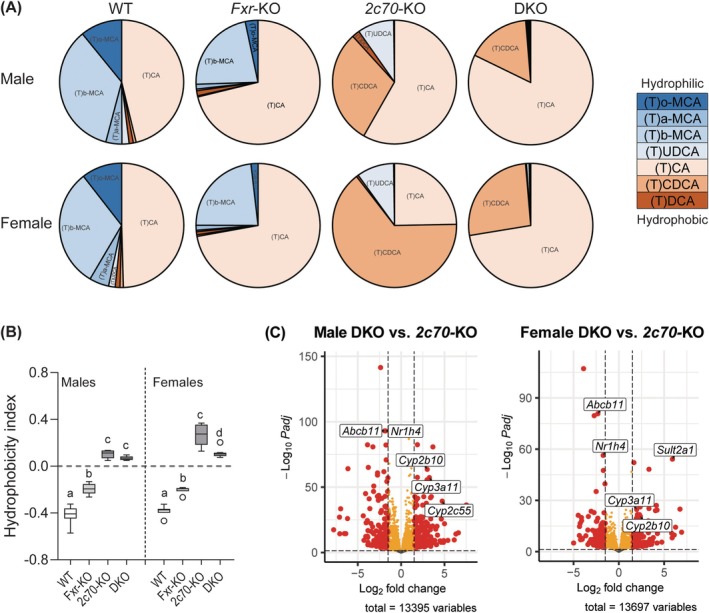
Increased 12α‐hydroxylated bile acids and transcriptional upregulation of detoxification‐related genes may contribute to reduced liver fibrosis in female DKO mice. (A) Biliary BA composition (*n* = 5–7 mice/group). (B) Hydrophobicity index of biliary BAs (*n* = 5–7 mice/group). Data are presented as Tukey box‐and‐whisker plots. (C) Volcano plots showing the differentially expressed genes (DEGs) DKO versus *2c70*‐KO mice in the livers of male mice (left) and female mice (right). Each point represents a gene, with significant DEGs (log_2_ fold change > 1 and FDR < 0.05). Groups with the same letters are not significantly different, whereas groups with different letters differ significantly (*p* < 0.05), assessed using the Kruskal‐Wallis H test with Conover post hoc comparisons. BA, bile acids; WT, wild type; Het, heterozygous; KO, knockout; DKO, double knockout; *2c70*, *Cyp2c70*.

In WT mice, over 40% of biliary BAs were MCAs, while the remaining part was mostly represented by 12α‐hydroxylated TCA (Figure 7A; Table S3). In line with previous observations [18], the BA composition shifted to a larger proportion of TCA with a concomitant reduction in TMCAs in *Fxr*‐KO mice, resulting in a more hydrophobic biliary BA composition (Figure 7B). TMCAs were absent in *Cyp2c70*‐KO mice, while non‐12α‐hydroxylated TCDCA was abundantly present leading to a positive hydrophobicity index (HI; Figure 7A,B), demonstrating a hydrophobic BA composition particularly in females. Similar to *Fxr*‐KO mice, TCA was the dominant BA in bile of DKO mice, reducing the HI compared to *Cyp2c70*‐KO mice, which was most obvious in females. In addition, the hepatic transcriptomes indicated significant upregulation of genes involved in BA detoxification in male as well as female DKO vs. *Cyp2c70*‐KO mice. Upregulated genes included putative BA hydroxylation genes like *Cyp2b10*, *Cyp3a11*, and *Cyp2c59*, and sulfonation genes (Figure 7C, S7). Together, these data suggest that increased production of 12α‐hydroxylated BAs and activation of BA detoxification contribute to a less hydrophobic and less cytotoxic BA profile which, in turn, may limit the development of liver fibrosis in female DKO mice.

## 4 Discussion

In this study, we generated a whole‐body *Fxr/Cyp2c70*‐DKO mouse model to explore the impact of *Fxr*‐deficiency in the context of a human‐like BA composition and to evaluate whether DKO mice recapitulate the pathophysiological features observed in human FXR‐deficiency. Our findings show that the lack of *Fxr* in mice with a human‐like BA profile aggravates hallmarks of cholestasis as compared to *Fxr*‐ or *Cyp2c70*‐single knockouts, including dysregulation of BA metabolism, exacerbation of hepatocytic injury, and enhanced inflammation. Intriguingly, we found that depletion of *Fxr* in the context of the human‐like BA composition in *Cyp2c70*‐KO mice confers protection from liver fibrosis and ductular reactions that are characteristic for female *Cyp2c70*‐KO mice, which associate with decreased expression of genes involved in EMT in the livers of these mice. This unexpected effect is conceivably attributable to a modest but distinct reduction in the hydrophobicity of biliary BAs.

In sharp contrast to FXR‐deficient patients [3, 5, 6, 7, 8, 9], *Fxr*‐KO mice do not display a severe cholestatic phenotype with concomitant progressive liver pathology at early life stages [17, 18, 32, 33]. We hypothesized that *Fxr*‐KO mice are protected from severe liver injury because of the presence of murine‐specific hydrophilic BAs, i.e., MCAs, in their BA pools. By deleting the *Cyp2c70* gene, mice acquire a human‐like and hydrophobic BA pool, devoid of these presumably hepatoprotective MCAs [21]. Although DKO mice do indeed display a more severe cholestatic phenotype than *Fxr* single knockouts as will be discussed in more detail below, DKO mice grow normally without noticeable differences in appearance compared to WT and *Fxr*‐KO mice. The phenotype of *Fxr/Cyp2c70*‐DKO mice appears to be somewhat milder than that of *Fxr/Shp*‐DKO mice, which show rather severe early onset (3 weeks of age) cholestatic liver injury [33]. These differences may be attributed to FXR‐nonoverlapping functions of *Shp*, as the phenotype of *Shp*‐KO mice is also considerably milder than that of *Fxr* */* *Shp*‐DKO mice [32, 33]. However, like the *Fxr/Cyp2c70*‐DKO mice, also *Fxr/Shp*‐DKO mice do not show overall growth retardation up to 1 year of age despite impaired weight gain from approximately 4 months onwards [32].

Hallmarks of cholestasis are more prominent in *Fxr* */* *Cyp2c70*‐DKO than in *Fxr* single knockouts. Hence, DKO mice do show similarities to the disease phenotype reported in human FXR‐deficiency [3, 5]. Dysregulated BA metabolism is one key factor contributing to exacerbated cholestasis. In line with previous studies [17, 18, 24], our data show that the loss of *Fxr* increases BA synthesis due to reduced expression of *Shp* in the liver and *Fgf15* in the intestine, leading to derepression of *Cyp7a1* and *Cyp8b1* expression in *Fxr*‐KO and DKO mice. Notably, plasma C4 levels were significantly increased in male DKO mice compared to other genotypes, suggesting higher CYP7A1 enzyme activity and BA synthesis in these animals [3]. The increased relative abundance of TCA in bile may be partly explained by increased CYP8B1 expression due to the lack of FXR repression [34].

As a central BA homeostasis regulator, *Fxr* induces the expression of BSEP [2]. In line with prior studies [35, 36], expression of BSEP was markedly reduced in *Fxr*‐KO mice. Of note, in analogy to the situation reported in FXR‐deficient patients [3], canalicular BSEP was virtually absent in DKO mice. In addition to FXR, inflammatory signalling has been reported to impact *Bsep* gene expression. The inflammatory cytokine interleukin 1β (IL‐1β) has been shown to downregulate *Bsep* transcription in hepatocytes via a CXCR2‐dependent pathway [37]. IL‐1β increases mRNA expression and release of CXCR2 ligands CXCL1 and CXCL2 that inhibit the CDCA‐induced upregulation of *Bsep* expression. This inhibitory effect could be attenuated by a CXCR2 inhibitor. In the current study, mRNA expression of *Il‐1β* was upregulated in livers of male DKO mice and mRNA expression of *Cxcl1* (Figure S3) was upregulated in female DKO mice. Although we did not measure circulating IL‐1β and CXCL1 levels as effects are most likely local in these models, their increased gene expression suggests that, in addition to the loss of *Fxr*‐mediated activation, inflammation‐driven suppression of its gene expression may also contribute to the absence of BSEP in these mice. Intriguingly, both *Fxr*‐KO and DKO mice showed an impaired bile flow, while biliary BA secretion rates remained largely unaffected. BSEP is considered the major BA transporter present in human liver [38]. However, alternative transporters that have been suggested to be capable of mediating hepatobiliary BA secretion in mice, such as MDR1 [39], conceivably partially compensate for the reduced BSEP activity in the *Fxr*‐deficient mice.

We did observe a higher prevalence of hypercholanemia in male DKO mice as compared to other groups. While this study was being finalized, Mishima et al. reported that liver‐specific *Fxr*‐inactivation in humanized *Cyp2a12*/*Cyp2c70* double knockout mice did not affect serum BA levels (< 15 μM) [40]. Moreover, mice with combined hepatic and intestinal *Fxr* deletion (*Fxr* ^ΔL/ΔIN^), which show a phenotype that is comparable to whole‐body *Fxr*‐KO mice, had markedly elevated serum BA levels, whereas single‐tissue knockouts (*Fxr* ^ΔL^ or *Fxr* ^ΔIN^) did not show higher serum BAs than WT [41]. Together, these findings underscore the cooperative role of hepatic and intestinal *Fxr* in regulation of plasma BA levels.

Notably, the increased liver‐to‐body weight ratios observed in male and female DKO mice, as well as in female Cyp2c70‐KO mice, were primarily associated with enhanced regenerative activity, as indicated by increased hepatocyte proliferation (Figure 3F,G) and ductular reaction (Figure 5A,C). In addition, hepatocyte ballooning and steatosis were observed in male DKO livers (Figure S8), which likely contributed to their increased weights as well.

Our group has previously described that female, but not male, *Cyp2c70*‐KO mice display progressive cholangiopathy and fibrosis with age [21]. Interestingly, sex‐specific differences were also observed in DKO mice. While ductular reactions and liver fibrosis have been reported in both male (*n* = 9) and female (*n* = 6) PFIC5 patients [3, 5, 6, 7, 8, 9, 10, 11, 12], the limited published cases and the lack of reported pathological details in these patients do not allow comparisons of the sex differences observed in DKO mice with human PFIC5. In addition, it has to be noted that all patients died or underwent liver transplantation before the age of 1 year, making it unlikely that sex hormones affect disease progression in human PFIC5 patients to a meaningful extent. Introducing *Fxr*‐deficiency in female *Cyp2c70*‐KO mice appeared to shift liver pathology from a mainly cholangiocyte‐driven to a hepatocyte‐driven type, with less ductular reactions and fibrosis, but more hepatocyte damage in female DKO mice. It is conceivable that accumulation of human‐like, hydrophobic BAs inside hepatocytes aggravated hepatocyte damage in DKO mice given the increased BA synthesis and reduced BSEP presence in the canalicular membranes in these animals as compared to *Cyp2c70*‐KO mice. However, direct quantification of BA levels within hepatocytes is complex, given the high BA concentrations in bile canaliculi and bile ducts, which cannot be effectively removed from excised livers. Therefore, in our opinion, it is impossible to assess whether the alterations in BA synthesis and transporter expression in DKO mice translated into intracellular BA accumulation. The reduced fibrosis and ductular reactions, on the other hand, may be explained at least in part by enhanced BA hydroxylation and detoxification in DKO mice, resulting in a less cytotoxic biliary BA profile. Notably, *Cyp3a11* and *Cyp2c55* encode enzymes that have been proposed to produce tetrahydroxylated bile acids (THBAs) in mice [42] and the rather benign phenotype of *Bsep*‐KO mice has also been attributed to their ability to produce THBAs [16, 43, 44]. Moreover, BA sulfation is an important phase II detoxification pathway that enhances water solubility of BAs, promotes their renal elimination and reduces toxicity [45, 46, 47]. Both of these processes appeared to be upregulated in DKO mice as compared to *Cyp2c70*‐KO mice.

Besides FXR, xenobiotic receptors also contribute to regulation of detoxification genes [48]. Hydrophobic BAs, primarily LCAs, can activate nuclear receptors such as pregnane X receptor (PXR) [49] and constitutive androstane receptor (CAR) [50]. Wagner et al. demonstrated that CAR and PXR agonists stimulate BA detoxification pathways in bile duct‐ligated mice [51], highlighting their potentially protective activities under cholestatic conditions. Although CAR and PXR activation were not studied in detail in the current study, analysis of the RNAseq data using the Gene Transcription Regulation Database [52] revealed that target genes of the latter were significantly overrepresented in the genes that were differentially expressed between DKO and *Cyp2c70*‐KO mice in both males and females (data not shown). In addition to the highly expressed phase I metabolism CYP genes (*Cyp3a11*, *Cyp2b10*, and *Cyp2c55*) and sulfation genes (Figure S7) in DKO livers mentioned above, several additional PXR‐ and CAR‐target genes, including UGT family members *Ugt1a1* and *Ugt1a6*, as well as hepatic transporters *Abcc3*, *Abcc4*, and *Oatp2*, were significantly upregulated in DKO mice as compared to *Cyp2c70*‐KO controls (Figure S9).

The mechanisms responsible for fibrogenesis in *Cyp2c70*‐KO mice [21, 53] are yet to be fully clarified. The RNAseq analysis in the current study highlights the induction of EMT as a process that may contribute to the development of fibrosis in these mice. EMT has been proposed to promote liver fibrosis by providing mesenchymal features to epithelial cells, that is, hepatocytes and cholangiocytes [28, 29, 30, 54]. While going through EMT, driven by pro‐fibrotic cytokines such as TGF‐β [54], these cells acquire the ability to produce components of the extracellular matrix. We demonstrate that EMT consistently represents the most upregulated pathway in livers of female *Cyp2c70*‐KO mice compared to WT mice, which was associated with severe cholangiopathy. In addition, we found a marked downregulation of EMT‐related genes in female DKO mice compared to *Cyp2c70*‐KO mice, which coincided with markedly reduced fibrosis. However, despite that cells expressing the EMT marker *S100a4* were evidently present in female DKO livers, their limited numbers and the lack of co‐localization with fibrogenic (*Col1a1*+) cells suggest that these cells do not play a major role in fibrogenesis in *Cyp2c70*‐KO mice. Of note, the role of EMT in liver fibrosis remains a matter of debate and no universal EMT markers have been defined so far [55]. Future studies will be required to elucidate the mechanistic role of EMT in the progression of liver fibrosis under cholestatic conditions.

Taken together, we conclude that induction of *Fxr*‐deficiency in the context of a human‐like BA composition, that is, in *Cyp2c70*‐KO mice, promotes hepatocytic injury, which is most conceivably driven by a hydrophobic BA overload. This DKO model recapitulates several key features of human FXR‐deficiency, including a virtual absence of canalicular BSEP as well as substantial hepatocyte damage and hepatic inflammation, highlighting its potential as a valuable tool for PFIC and, potentially, BA‐induced hepatocellular carcinoma research. Unexpectedly, *Fxr* ablation in female *Cyp2c70*‐KO mice reduces the ductular reactions and fibrosis that are characteristic for these mice, thereby providing clues to define the biological processes driving fibrogenesis. Future studies should aim to explore the mechanistic details of these protective processes, which may help in advancing the treatment of cholangiopathic and cholestatic liver diseases.

## Author Contributions

Conceptualization: Henkjan J. Verkade, Jan Freark de Boer, Folkert Kuipers. Data acquisition: Hilde D. de Vries, Jinxiao Li, Milaine V. Hovingh, Niels L. Mulder, Rick Havinga, Vincent W. Bloks, Anna Palmiotti, Krisztina de Bruyn, Brecht Attema, Ellen Weersing, Martijn van Faassen. Formal analysis: Rachel E. Thomas, Kirill Ustyantsev, Eugene Berezikov. Drafting the manuscript: Hilde D. de Vries, Jinxiao Li. Critical revising: Jan Freark de Boer, Henkjan J. Verkade, Folkert Kuipers. All authors have agreed to the published version of the manuscript.

## Funding

Hilde D. de Vries is supported by internal UMCG‐grant PPP‐2022‐02. Jinxiao Li is supported by China Scholarship Council (no. 202207720013). Henkjan J. Verkade received grants from Albireo/Ipsen, Mirum, Rectify, C&W de Boer Stichting, European Society for Paediatric Gastroenterology, Hepatology and Nutrition (ESPGHAN) and from internal UMCG‐grant PPP‐2022‐02 (collaboration with Albireo; a collaboration project co‐financed by the Ministry of Economic Affairs and Climate Policy by means of the allowance made available by the Top Sector Life Sciences & Health to stimulate public‐private partnerships). Folkert Kuipers is supported by an unrestricted grant from the Noaber Foundation, Lunteren, The Netherlands.

## Conflicts of Interest

Henkjan J. Verkade is the consultant of Albireo/Ipsen, Intercept, Mirum, Orphalan, ProQR, and Vertex. All other authors disclose no conflicts of interest.

## Supporting information


**Table S1:** Primer sequences.
**Table S2:** Primary probe sequences used in RNA in situ hybridization.
**Table S3:** Biliary bile acid composition (percentage).

**Data S2:** RNAseqData_Females.

**Data S3:** RNAseqData_Males.

## Data Availability

The data that support the findings of this study are available from the corresponding author upon reasonable request.
