# Supplementary material for: Farnesoid x Receptor Deficiency Promotes Hepatocytic Injury in Cyp2c70‐Deficient Mice With a Human‐Like Bile Acid Composition
Source: Liver Int. 2026 Apr 9;46(5):e70632. doi: 10.1111/liv.70632 (PMC13064423; doi:10.1111/liv.70632)
Supplement: Supplementary file 1 — Table S1: Primer sequences. Table S2: Primary probe sequences used in RNA in situ hybridization. Table S3: Biliary bile acid composition (percentage). [file LIV-46-0-s003.docx]

**Supplemental data with**

**Farnesoid x receptor deficiency promotes hepatocytic injury in *Cyp2c70*-deficient mice with a human-like bile acid composition**

Hilde D. de Vries^1*^, Jinxiao Li^1*^, Kirill Ustyantsev^2^, Milaine V. Hovingh^1^, Niels L. Mulder^1^, Rick Havinga^1^, Anna Palmiotti^1^, Krisztina de Bruyn^3^, Brecht Attema^2^, Ellen Weersing^2^, Rachel E. Thomas^4^, Vincent W. Bloks^1^, Eugene Berezikov^2^, Martijn van Faassen^3^, Henkjan J. Verkade^1^, Jan Freark de Boer^1,3,#,‡^, Folkert Kuipers^2,3,#,‡^

**Affiliations**

^1^Department of Pediatrics, University of Groningen, University Medical Center Groningen (UMCG), Groningen, The Netherlands.

^2^European Research Institute for the Biology of Ageing (ERIBA), University of Groningen, University Medical Center Groningen (UMCG), Groningen, The Netherlands.

^3^Department of Laboratory Medicine, University of Groningen, University Medical Center Groningen (UMCG), Groningen, The Netherlands.

^4^Royal GD, Deventer, The Netherlands.

*^#^ These authors equally contributed to this study

^‡^Corresponding Author: f.kuipers@umcg.nl and j.f.de.boer@umcg.nl

**Content**

Supplemental Methods

Supplemental Tables

Supplemental Figures

**Supplemental Methods**

**Animals and Sample Collection**

All mice were housed individually at 20-22°C with a 12-hour light/dark cycle. Mice had *ad libitum* access to water and a standard irradiated chow diet (Ssniff^®^ 1554R/M-H maintenance diet). Genotyping was performed via PCR on genomic DNA extracted from ear biopsies, using primers listed in Table S1. PCR products were separated by 3% agarose gel electrophoresis and visualized using a gel imaging system (Bio-Rad).

For bile collection, 12- to 15-week-old mice were anesthetized via intraperitoneal injection of Hypnorm (fentanyl/fluanisone 1 mL/kg) and diazepam (10 mg/kg). The common bile duct was ligated, and bile was collected for 30 minutes. Bile flow rate per minute was calculated based on the bile weight. Mice were euthanized by cardiac puncture under isoflurane anesthesia, followed by cervical dislocation. Blood was centrifuged at 8000 rpm for 10 minutes at 4°C to obtain plasma, which was stored at -80°C until further analyses.

**Gene Expression**

Frozen liver tissues were pulverized under liquid nitrogen and transferred to pre-cooled 2 mL microcentrifuge tubes. A stainless steel bead (QIAGEN) and 1 mL TRI-Reagent (Sigma-Aldrich, St. Louis, MO) were added to each tube, and samples were homogenized at 50 Hz for 2 min using a QIAGEN Tissuelyser (Venlo, the Netherlands). Chloroform (200 μL) was added for phase separation, and the mixtures were centrifuged at 12000xg for 15 min at 4 °C. The upper (aqueous) phase was transferred to a new microcentrifuge tube, and RNA was precipitated by adding 500 μL of isopropanol. After incubation for 10 min at room temperature, samples were centrifuged at 12000xg for 10 min at 4 °C to form RNA pellets. Pellets were washed with 75% ethanol, air-dried shortly, and dissolved in RNAase-free water.

Reverse transcription was performed using 1 μg of total RNA, Moloney Murine Leukemia Virus Reverse Transcriptase (Invitrogen), and Random Nonamers (Sigma-Aldrich). Quantitative RT-PCR was performed on a QuantStudio 7 System (Applied Biosystems, Foster City, CA) using either TaqMan primer-probe combinations (Applied Biosystems, Foster City, CA) or SYBR green mastermix (Roche Diagnostics). Primers sequences used in this study are listed in Table S1.

**Western Blotting**

Total protein was extracted from liver homogenates (10%, w/w) using lysis buffer (50 mM Tris-HCl, pH 7.4, 150 mM NaCl, 1 mM EDTA), supplemented with protease inhibitor (Roche, Switzerland) and 200 μM PMSF. Protein concentrations were quantified using the Pierce BCA protein assay kit, according to the manufacturer protocol (Thermo Fisher Scientific, Waltham, MA). Samples were incubated at 37 °C for 20 min, and 45 μg of protein per sample was separated by 5% SDS-PAGE gel electrophoresis and transferred to nitrocellulose membranes (0.22 μm). Membranes were blocked with either 5% non-fat milk in Tris-buffered saline containing 0.1% Tween-20 (TBST; for BSEP) or 5% bovine serum albumin in TBST buffer (for HSP90) for 1 hour and subsequently incubated overnight at 4°C with primary antibodies against BSEP (1:2000; BosterBio, PB9414) and against HSP90 (1:2000; Cell Signaling, #4874) overnight at 4°C. After primary antibody incubation, membranes were washed with TBST and then incubated with HRP-conjugated secondary antibody goat anti-rabbit IgG (1:2000; DAKO, P0448) for 1 hour at room temperature. Signals were developed using SuperSignal West Femto substrate (Thermo Fisher Scientific, Waltham, MA). Membranes were imaged with the ChemiDoc MP Imaging System (Bio-Rad Laboratories, California, USA) and quantified using Image Lab (v5.2.1, Bio-Rad Laboratories, California, USA).

**Histological Analysis** Liver H&E slides were analyzed in a blinded manner by a board certified veterinary pathologist. Initial screening of the samples was performed at 10x and the range and extent of the different types of the pathology were identified, so that these could be scored. Counting/scoring of these features was performed in 10 randomly selected high-powered fields (hpf, 20x) using a scale of 0 = absent/minimal, 1 = mild, 2 = moderate, 3 = severe. Any form of liver damage (inflammation or metabolic disturbance) can result in hepatocyte death through necrosis, apoptosis or another form of cell death which are not morphologically distinguishable, collectively referred to here as single-cell death. A mild degree of single-cell death constituted 0-2 hypereosinophilic cels with nuclear fading in each hpf, moderate cell death involved 2-5 dead cells in each hpf, and severe SCD was scored only when > 5 necrotic cells were seen in each hpf. Pigmented macrophages are indicative of phagocytosis of lipid-iron-containing materials and are associated with cellular damage; these were scored according to Kleiner et al.[1] as none-rare = 0, many = 1. Ballooning cells were occasionally identified and characterized by marked hypertrophy (compared to regular hepatocytes due to microvesicular vacuolation) with cytoplasmic changes +/- a centrally positioned nucleus that was deformed/obscured by the vacuoles; 0 = absent, 1 = few (rare but definite ballooned hepatocytes as well as case that are diagnostically borderline), 2 = frequent (according to Kleiner et al[1]). Steatosis was scored routinely whereby < 5% of cells affected = minimal/absent, < 33% cells affected = mild, > 66% cells affected = severe.

**Immunofluorescent Staining**

Deparaffinized liver sections (4 µm) were subjected to heat-induced antigen retrieval by boiling in 10 mM citrate buffer (pH 6.0) for 20 min and were subsequently cooled down for 15-20 min. Liver sections were washed by PBS and incubated with 5% goat normal serum (DAKO; X0907) for 30 min to block unspecific staining. These sections were then incubated with primary antibody against BSEP (1:1000; BosterBio, PB9414) for 1 hour at room temperature. After washing with PBS, sections were incubated with secondary antibody Alexa Fluor 568-conjugated goat anti-rabbit IgG (1:1000; Invitrogen, A11011) for 30 min. DAPI (1:100; Roche, #10236276001) was used to counterstain for cell nuclei. Sections were mounted with fluorescence mounting medium (Dako, S3023). Images were obtained by using a Leica DMI6000B fluorescence microscope (Leica Microsystems, Wetzlar, Germany) equipped with a Leica DFC365 FX camera.

**Immunohistochemistry Staining**

Deparaffinized liver sections (4 µm) were subjected to either enzymatic antigen retrieval (0.1% pronase; Roche, #10165921001) or heat-induced antigen retrieval (Tris-EDTA buffer, pH 9.0 or 10 mM citrate buffer, pH 6.0). Liver sections were washed with PBS and incubated with 3% hydrogen peroxide for 10 min to block endogenous peroxidase. The sections were washed with PBS and incubated with 5% goat normal serum (DAKO; X0907) for 30 min at room temperature in a humidity chamber. After removing the blocking solution, sections were incubated with primary antibody overnight at 4°C. The following primary antibodies were used: CK19 (1:1000; Abcam, ab52625), Ki-67 (1:1000; Abcam, ab15580), and F4/80 (1:200; Bio-Rad, MCA497). After washing with PBS, sections were incubated with secondary antibody for 30 min at room temperature. The following peroxidase-conjugated secondary antibodies were used: goat-anti-rabbit HRP (1:100; DAKO, P0448), goat anti-rat IgG antibody (1:125; Vector, BA-9400). Immune complexes were visualized using DAB substrate kit (Thermo Scientific, # 34002). Sections were counterstained with hematoxylin, dehydrated with series of ethanol, and sealed with mounting medium (Sigma-Aldrich, # 03989). Images were acquired using a Hamamatsu NanoZoomer (Hamamatsu Photonics, Almere, the Netherlands).

**RNA Fluorescence In Situ Hybridization (FISH)**

Paraffin sections were hybridized using OneSABER tyramide signal amplification (TSA) FISH with *Col1a1* and *S100a4* probes (Integrated DNA Technologies; sequences in Table S2) according to previously described protocols[2]. Briefly, sections (5 µm thickness) were deparaffinized, followed by blocking with Bloxall solution (Vector, SP-6000-100) to eliminate potential endogenous alkaline phosphatase (AP) and peroxidase activity. Slides were next placed in Sequenza® Slide Racks and used for subsequent procedures. Slides were washed with PBS-0.1% Tween and incubated with TE buffer for 1 h at 70°C in a hybridization oven, followed by treatment with proteinase K (20 μg/mL in PBS-0.1% Tween; Sigma-Aldrich, EO0491) for 10 min at 37°C. Hybridization was conducted using gene-specific SABER probes (S100a4-p27, Col1a1-p28) in Hyb buffer overnight at 50°C in a hybridization oven. Post-hybridization, slides were washed with WashHyb and 2×SSCTw buffers. Secondary oligonucleotide probes complementary to the SABER concatemer sequences were hybridized for 1 h at 42°C in PBS-0.1% Tween containing 10% dextran sulfate. After washing, slides were blocked with 5% horse serum and 5% Western Blocking Reagent for 30 min at room temperature, followed by incubation with anti-DIG/FITC-POD antibodies (1:2000 dilution) for 45 min at 37°C. Fluorescent signal development was performed using fluorescent tyramides to detect *S100a4* (CF647 for *S100a4*, 1:250 in TSA development buffer; Biotium #96022) and *Col1a1* (CF568, 1:1000 in TSA development buffer; Biotium #92173) by incubation in the dark for 10 min at room temperature. Fluorescent signal development was performed sequentially for each probe; slides were treated with sodium azide (0.1 M, 30 min at room temperature) after development of the first probe to inactivate residual peroxidase activity. The blocking, antibody incubation, and signal development steps were then repeated for the second probe. Sections were counterstained with DAPI (2 µg/ml, 30 min at room temperature) and mounted with Vectashield Vibrance Antifade Mounting Medium (Vector, H-1700). Confocal images were captured on a Leica SP8x microscope (63x/1.4 oil objective) at the UMCG Microscopy and Imaging Center (UMIC) and processed by ImageJ (v1.54d, National Institutes of Health, Bethesda, MD).

**RNA Library Preparation and Sequencing**

The RNA yield was measured using the NanoDrop Microvolume Spectrophotometer (ThermoFisher Scientific). The RNA-Seq libraries were prepared according to the Smart-3SEQ protocol[3] using 100 ng of total RNA per sample as input. The libraries were pooled at the ERIBA Research Sequencing Facility (Groningen, the Netherlands) and sequenced on Illumina NovaSeq platforms using a paired-end 150 bp sequencing strategy at Novogene Co. Ltd. Europe.

**Reference**

1. Kleiner DE, Brunt EM, Van Natta M, Behling C, Contos MJ, Cummings OW, et al. Design and validation of a histological scoring system for nonalcoholic fatty liver disease. Hepatol Baltim Md. 2005;41:1313–21. https://doi.org/10.1002/hep.20701

2. Ustyantsev K, Stranges M, Volpe FG, de Boer JF, Kuipers F, Mouton S, et al. One probe fits all: a highly customizable modular RNA in situ hybridization platform expanding the application of SABER DNA probes. Dev Camb Engl. 2025;152:dev204775. https://doi.org/10.1242/dev.204775

3. Foley JW, Zhu C, Jolivet P, Zhu SX, Lu P, Meaney MJ, et al. Gene expression profiling of single cells from archival tissue with laser-capture microdissection and Smart-3SEQ. Genome Res. 2019;29:1816–25. https://doi.org/10.1101/gr.234807.118

**Supplemental Tables**

**Table S1: Primer sequences**

| Gene | Species | Sequence | |
| --- | --- | --- | --- |
| *Cyp2c70* | Mus | forward | 5’-CGTGGCAGAGGGAAGCTC-3’ |
|  |  | reverse | 5’-GGGTGGAAGGAAGTCACTGA -3’ |
| *Fxr/Nr1h4* | Mus | forward | 5′-GTTGTAGTGGTACCCAGAGGCCCTG-3′ |
|  |  | reverse | 5′-TATGCTAACAGA ACACGCGGCAGGC-3′ (WT allele) |
|  |  | reverse | 5′-GGGTGGGATTAGATA AATGCCTGCTCT-3′ (mutant allele) |
| *Cyclophilin G* | Mus | forward | 5’-CAGATCGAGGGATCGATTCAG-3’ |
|  |  | reverse | 5’-TCACCACTTGACACCCTCATTC-3’ |
|  |  | probe | 5’-CTCCTCCACATTGGAGACAAGAGATGCA-3’ |
| *Fxr/Nr1h4* | Mus | forward | 5’-GAGGGCTGCAAAGGT TTC TTC-3’ |
|  |  | reverse | 5’-ACTTCCTGCGCATGTACATGTC-3’ |
|  |  | probe | 5’-CCGTTCTTACACTTGTACACGGCGTTCTTG-3’ |
| *Shp/Nr0b2* | Mus | forward | 5’-AAGGGCACGATCCTCTTCAA-3’ |
|  |  | reverse | 5’-CTGTTGCAGGTGTGCGATGT-3’ |
|  |  | probe | 5’-ATGTGCCAGGCCTCCGTGCC-3’ |
| *Cyp7a1* | Mus | forward | 5’-CAGGGAGATGCTCTGTGTTCA-3’ |
|  |  | reverse | 5’-AGGCATACATCCCTTCCGTGA-3’ |
|  |  | probe | 5’-TGCAAAACCTCCAATCTGTCATGAGACCTCC-3’ |
| *Cyp8b1* | Mus | forward | 5’-AAGGCTGGCTTCCTGAGCTT-3’ |
|  |  | reverse | 5’-AACAGCTCATCGGCCTCATC-3’ |
|  |  | probe | 5’-CGGCTACACCAAGGACAAGCAGCAAG-3’ |
| *Fgf15* | Mus | forward | 5’-GCCATCAAGGACGTCAGCA-3’ |
|  |  | reverse | 5’-CTTCCTCCGAGTAGCGAATCAG-3’ |
|  |  | probe | 5’-CGCTCATGCAGAGGTACCGCACG-3’ |

**Table S2: Primary probe sequences used in RNA *in situ* hybridization**

| Gene | Probe_ID | Sequence 5’-3’ with p27 3’ initiator sequence (tttCATCATCAT) |
| --- | --- | --- |
| *Col1a1* | 1 | CACATACAAAATAGGTACAGAGTCTCTTGTTTCCTCCCAGCCtttCATCATCAT |
|  | 2 | GGGTTTCAGAAGAAAGAGAGAGATCAGATAGGCCCAGGAtttCATCATCAT |
|  | 3 | GTCCCCGCACTCTCTTCTCCCTTGTTAAATAGCACCTTtttCATCATCAT |
|  | 4 | ACATTTGCTGGTCTAGGGAGCATCTCAGCTTCTATAGCtttCATCATCAT |
|  | 5 | CCACCCCTTCACAGAGATGTAGCACCTTTGATACCAAAtttCATCATCAT |
|  | 6 | ACACACAAAGACAAGAACGAGGTAGTCTTTCAGCAACACAtttCATCATCAT |
|  | 7 | GGAGATGCCAGATGGTTAGGTTCCTTCAACAGTCCAAGtttCATCATCAT |
|  | 8 | CTTTGTTTGCTGGGCAGACAATGCATTATTTCCTGTGTCTtttCATCATCAT |
|  | 9 | ACTTATACCCACATAGGTCTTCAAGCAAGAGGACCAAGCtttCATCATCAT |
|  | 10 | GGGGAAATTGAGTTTGGGTTGTTCGTCTGTTTCCAGGGtttCATCATCAT |
|  | 11 | TCTTGGTGGTTTTGTATTCGATGACTGTCTTGCCCCAAtttCATCATCAT |
|  | 12 | CATGCTCTCTCCAAACCAGACGTGCTTCTTTTCCTTGGtttCATCATCAT |
|  | 13 | CTTTAACACCAGTATCACCAGGTTCACCTTTCGCACCAtttCATCATCAT |
|  | 14 | TTTTCATCATAGCCATAGGACATCTGGGAAGCAAAGTTTCCTtttCATCATCAT |
|  | 15 | TCCCTCGACTCCTACATCTTCTGAGTTTGGTGATACGTtttCATCATCAT |
|  | 16 | GCAGATACAGATCAAGCATACCTCGGGTTTCCACGTCTtttCATCATCAT |
|  | 17 | CCATTGTGTATGCAGCTGACTTCAGGGATGTCTTCTTGtttCATCATCAT |
|  | 18 | ACATGTCTAGACCCTAGACATGTAGACTCTTTGCGGCTtttCATCATCAT |
| *mS100a4* | 1 | AACCTTTTATTGAATTTGCTCAGCACTGTGCACATGTGCtttCATCATCAT |
|  | 2 | AGTAAGGCACTATGCTCACAGCCAACAGGGAAGATCCtttCATCATCAT |
|  | 3 | TGGCAAACTACACCCCAACACTTCATCTGAGGAGTCTtttCATCATCAT |
|  | 4 | CCTTATCTGGGCAGCCCTCAAAGAATTCATTGCACATtttCATCATCAT |
|  | 5 | CAATGCAGGACAGGAAGACACAGTACTCCTGGAAGTCtttCATCATCAT |
|  | 6 | CATTGTCCCTGTTGCTGTCCAAGTTGCTCATCACCTTtttCATCATCAT |
|  | 7 | CCCTGGTCAGTAGCTCCTTGAGCTCTGTCTTGTTCAGtttCATCATCAT |
|  | 8 | ACTTGTCACCCTCTTTGCCTGAGTATTTGTGGAAGGTtttCATCATCAT |
|  | 9 | TTGCCATGGTAACCGTTGAGACCAGACCAAGAGAGAGtttCATCATCAT |

**Table S3: Biliary bile acid composition (percentage)**

|  | **Male** | | | | **Female** | | | |
| --- | --- | --- | --- | --- | --- | --- | --- | --- |
|  | **WT** | ***Fxr*-KO** | ***2c70*-KO** | **DKO** | **WT** | ***Fxr*-KO** | ***2c70*-KO** | **DKO** |
| (T)CA | 46.2% | 71.2% | 58.3% | 82.2% | 49.6% | 72.2% | 24.7% | 72.4% |
| (T)CDCA | 0.7% | 0.3% | 30.0% | 16.5% | 0.9% | 0.4% | 64.8% | 26.3% |
| (T)DCA | 1.1% | 1.3% | 2.0% | 0.2% | 1.2% | 0.8% | 0.5% | 0.2% |
| (T)UDCA | 1.9% | 0.4% | 9.5% | 0.3% | 1.7% | 0.4% | 9.7% | 0.7% |
| (T)a-MCA | 4.2% | 1.2% | 0.0% | 0.3% | 5.1% | 1.3% | 0.0% | 0.1% |
| (T)b-MCA | 35.0% | 22.2% | 0.2% | 0.2% | 31.0% | 23.1% | 0.2% | 0.1% |
| (T)o-MCA | 10.9% | 3.3% | 0.0% | 0.3% | 10.5% | 1.8% | 0.0% | 0.3% |

Data are presented as mean values. N = 5-7 mice/group.

**Supplemental Figures**

**
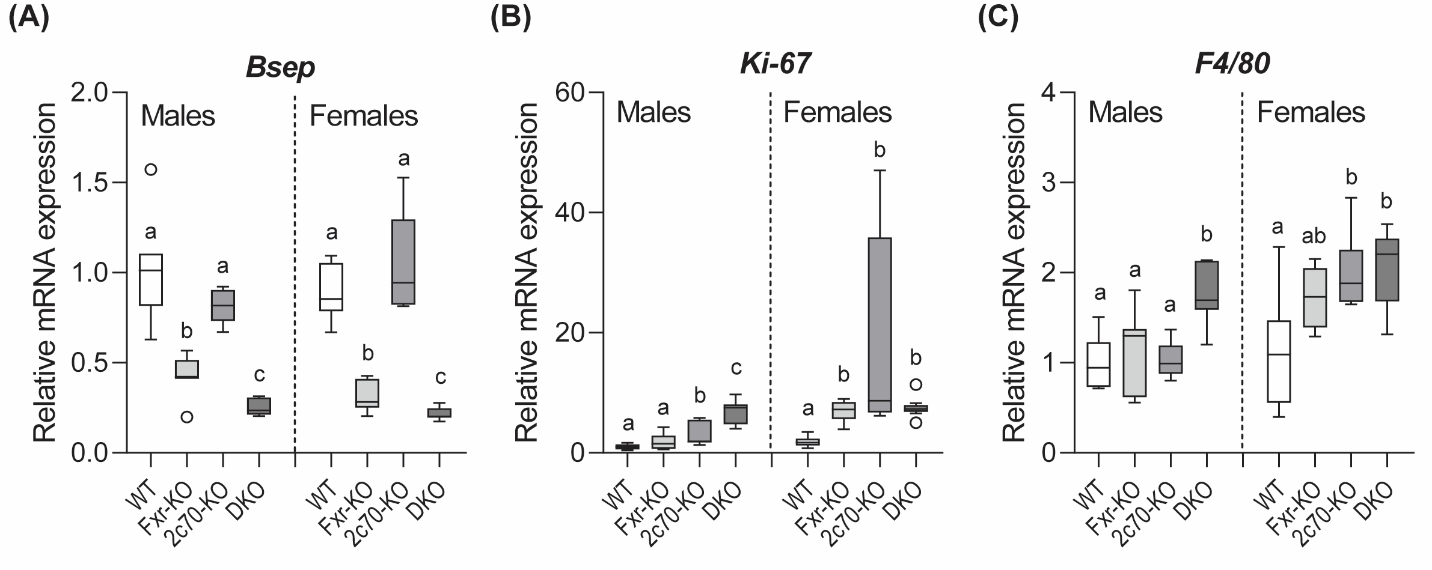
**

**Supplemental Figure 1** (A-C) Hepatic mRNA expression of *Bsep*, *Ki-67*, and *F4/80*. Gene expression levels were quantified using normalized counts data determined by RNA-sequencing (n = 6-9 mice/group). Data are presented as Tukey box-and-whisker plots. Groups with the same letters are not significantly different, whereas groups with different letters differ significantly (*P* < 0.05), assessed using the Kruskal-Wallis H test with Conover post hoc comparisons. WT, wild type; Het, heterozygous; KO, knockout; DKO, double knockout; *2c70*, *Cyp2c70*.

**Supplemental Figure 2** Plasma bile acid composition in male and female WT, *Fxr*-KO, *Cyp2c70*-KO and DKO mice. N = 6-9 mice/group.


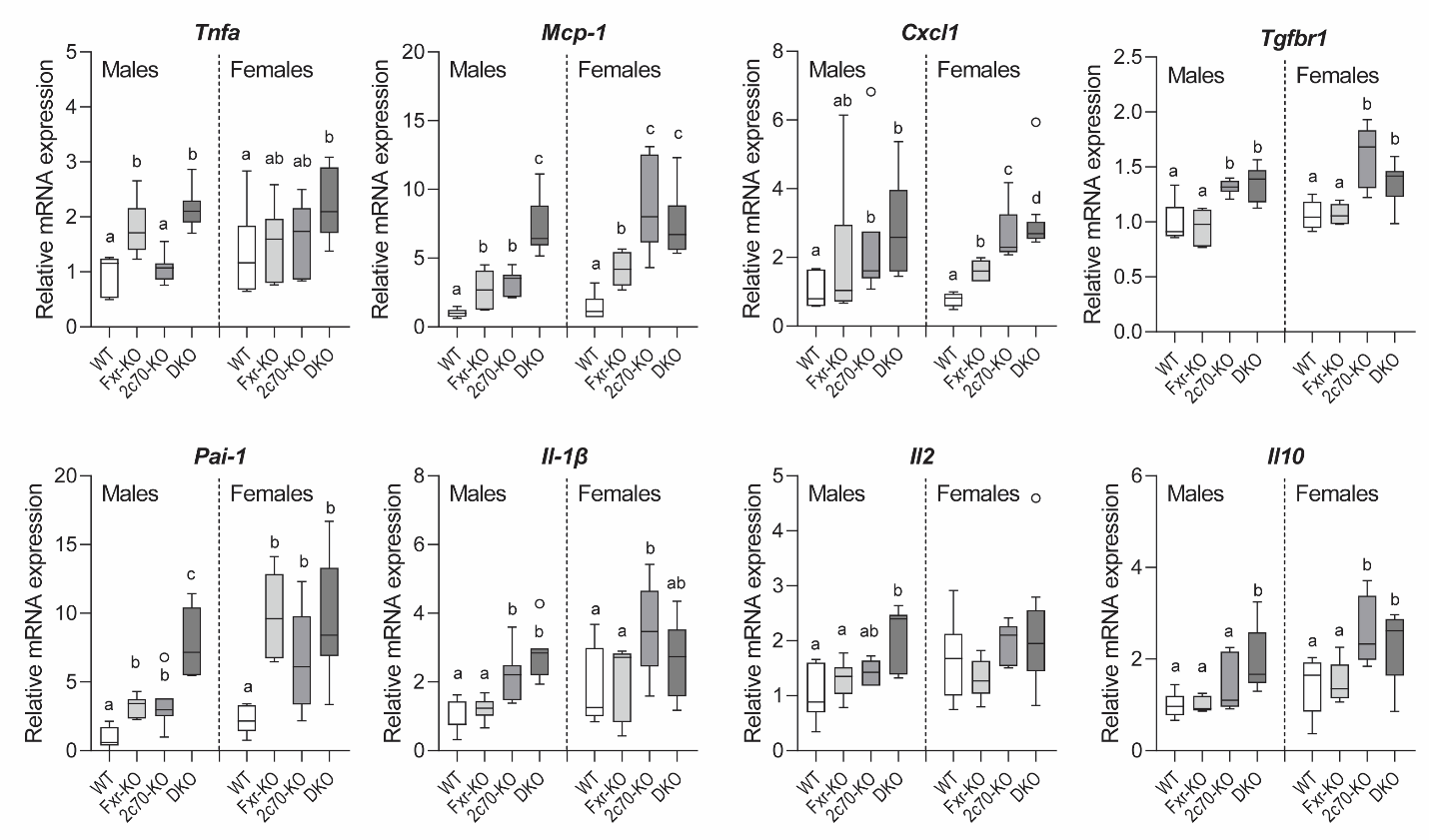


**Supplemental Figure 3** Hepatic mRNA expression of inflammation-related genes. Gene expression levels were quantified using normalized counts data determined by RNA-sequencing (n = 6-9 mice/group). Data are presented as Tukey box-and-whisker plots. Groups with the same letters or without letters are not significantly different whereas groups with different letters differ significantly (*P* < 0.05), assessed using the Kruskal-Wallis H test with Conover post hoc comparisons. WT, wild type; Het, heterozygous; KO, knockout; DKO, double knockout; *2c70*, *Cyp2c70*.

**
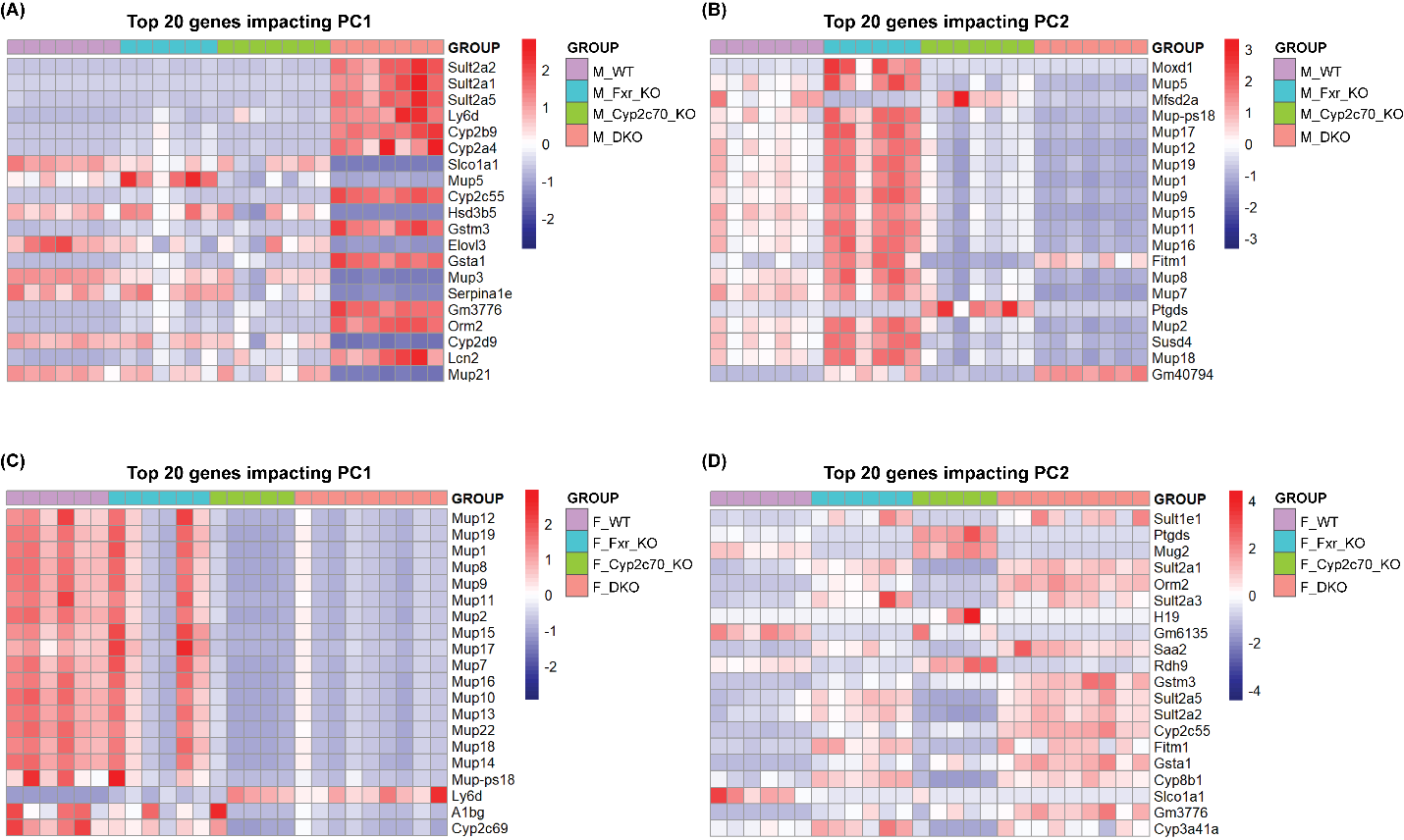
**

**Supplemental Figure 4** Heatmap for genes with the strongest positive and negative loadings on principal component 1 (PC1) in (A) males and (C) females, as well as on principal component 2 (PC2) in (B) males and (D) females, ranked in decreasing order of the absolute loading value of each gene.


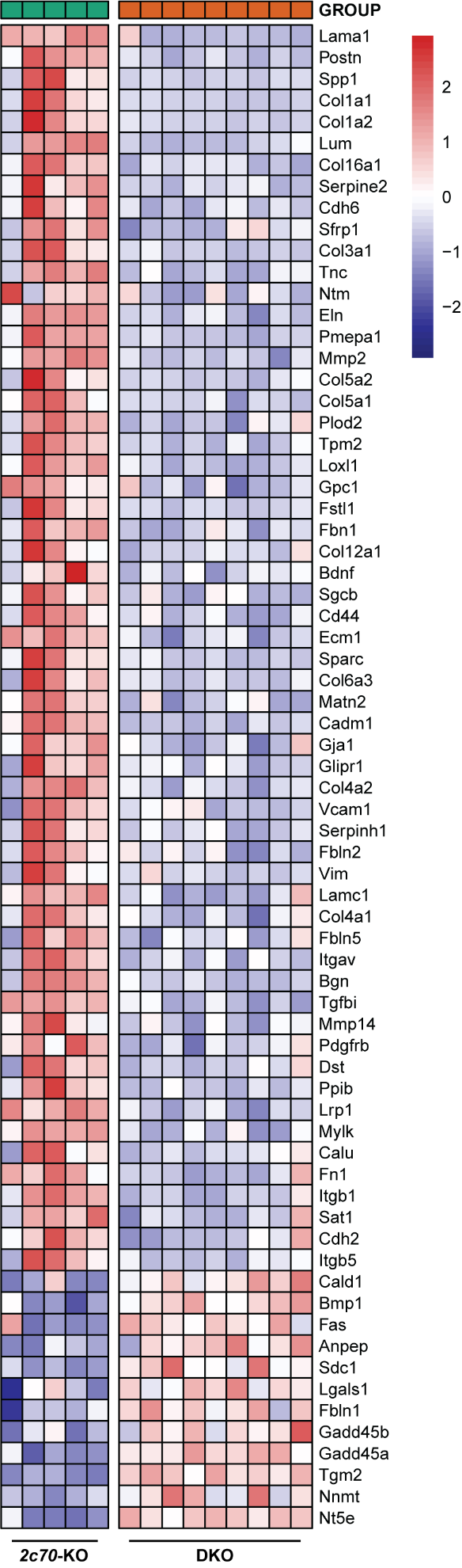
**Supplemental Figure 5** Heatmap showing genes from the “Epithelial-Mesenchymal Transition” Hallmark gene set that were significantly altered (FDR-adjusted *P* < 0.05) in female DKO versus *Cyp2c70*-KO mice, ranked in ascending order of log2 fold-change values.


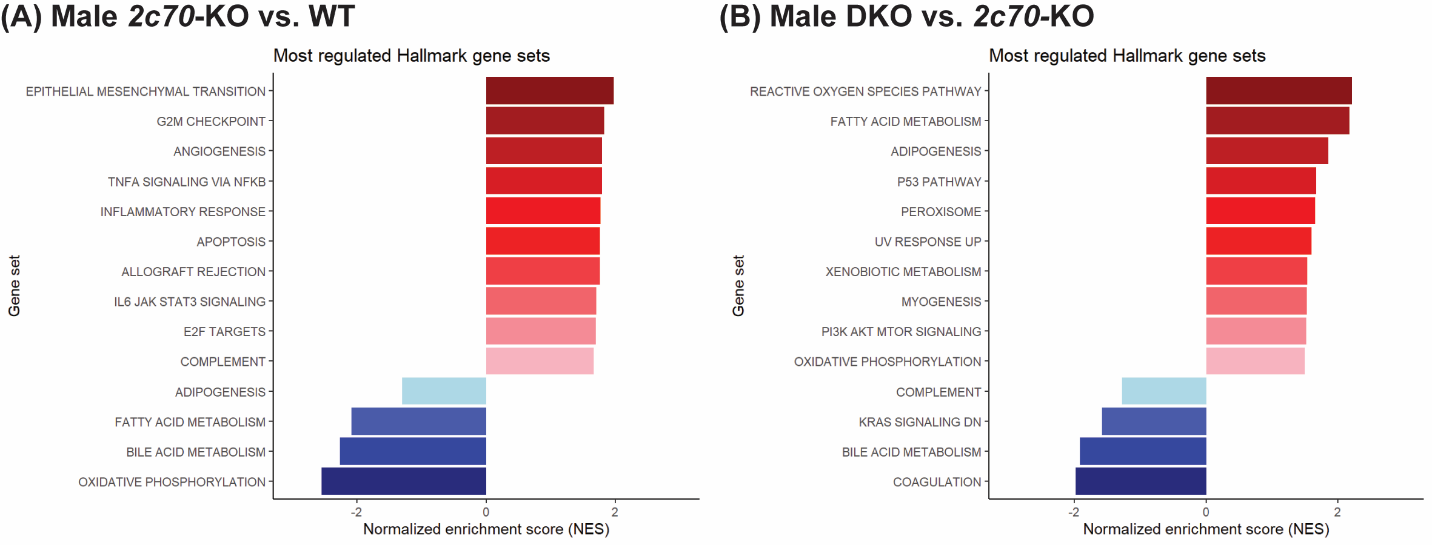


**Supplemental Figure 6** (A) Top enriched upregulated and downregulated Hallmark gene sets identified by GSEA in male *Cyp2c70*-KO mice versus WT controls, ranked by stat values. All displayed gene sets had FDR-adjusted *P*-value < 0.05. (B) Top enriched upregulated and downregulated Hallmark gene sets identified by GSEA in male *Cyp2c70*-KO mice versus WT controls, ranked by stat values. All displayed gene sets had FDR-adjusted *P*-value < 0.05, except the COMPLEMENT gene set (FDR = 0.13).

**
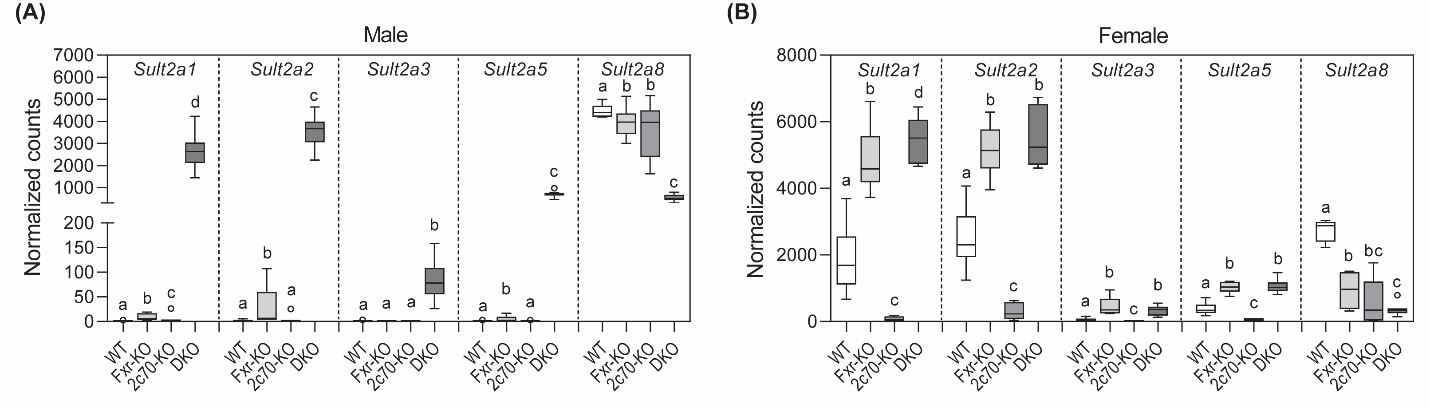
**

**Supplemental Figure 7** Hepatic mRNA expression of *Sult2a* genes in (A) males and (B) females. Gene expression levels were quantified using normalized counts data determined by RNA-sequencing (n = 6-9 mice/group). Data are presented as Tukey box-and-whisker plots. Groups with the same letters are not significantly different whereas groups with different letters differ significantly (*P* < 0.05), assessed using the Kruskal-Wallis H test with Conover post hoc comparisons. WT, wild type; Het, heterozygous; KO, knockout; DKO, double knockout; *2c70*, *Cyp2c70*.


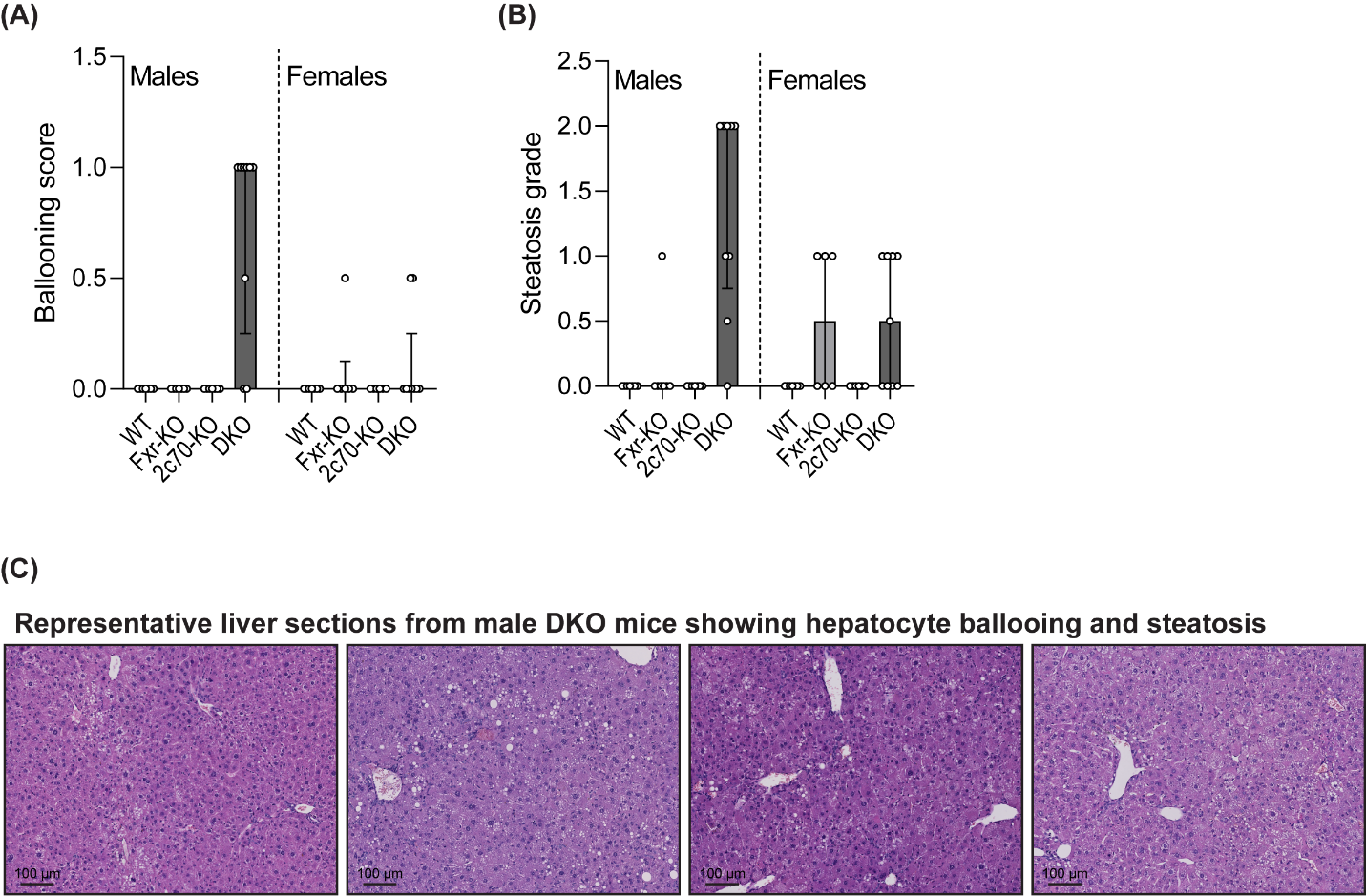
**Supplemental Figure 8** (A) Hepatocyte ballooning scores and (B) steatosis grades, assessed by a veterinary pathologist on H&E-stained liver sections (n = 6-9 mice/group). Data are presented as bar plots showing median with interquartile range. (C) Representative H&E staining of liver sections from four individual male DKO mice showing hepatocyte ballooning and steatosis. Scale bars: 100 µm. WT, wild type; KO, knockout; DKO, double knockout; *2c70*, *Cyp2c70*.

**
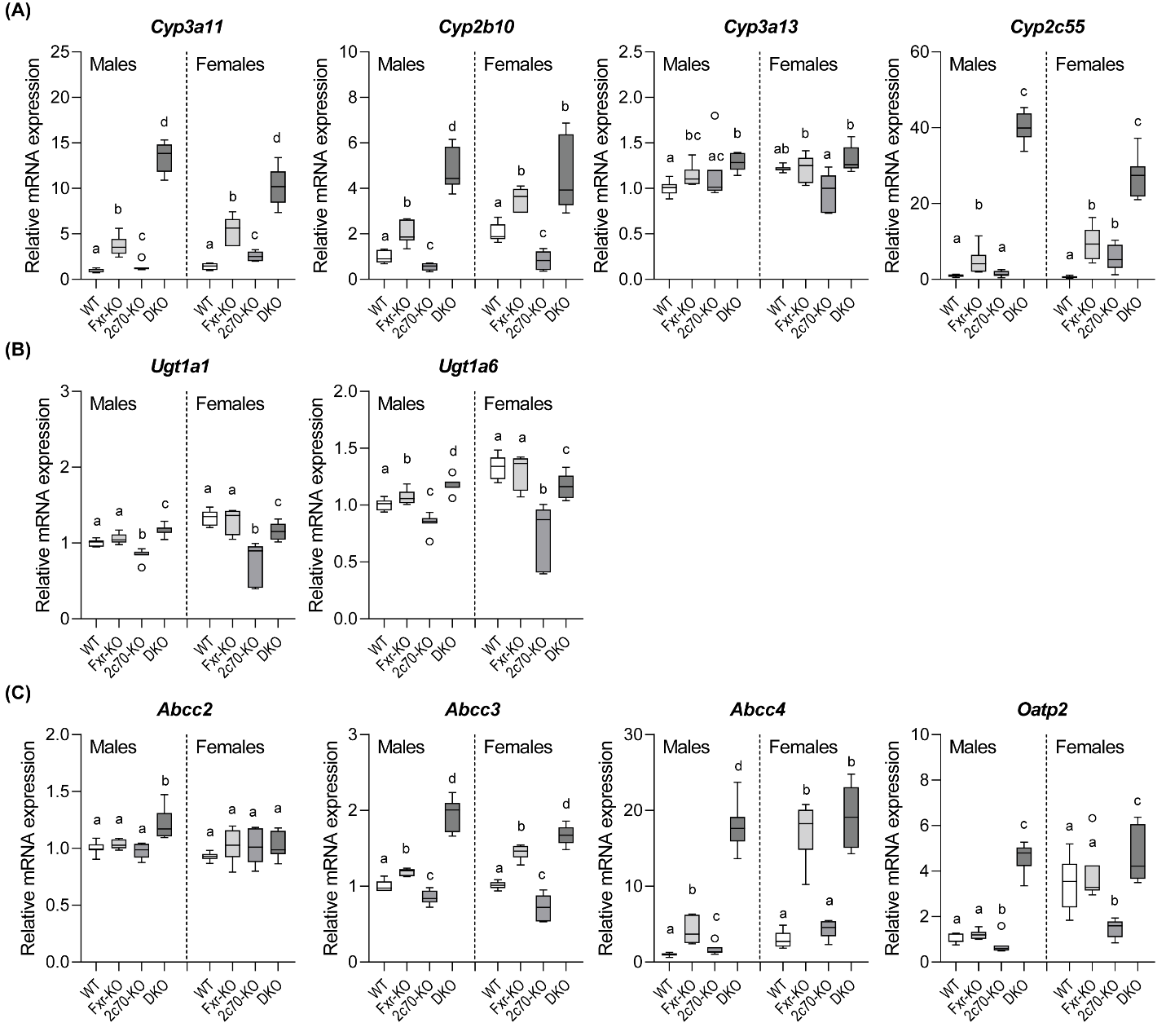
**

**Supplemental Figure 9** Hepatic mRNA expression of PXR- and CAR- target genes involved in xenobiotic metabolism. (A) Phase I metabolism (CYPs) genes *Cyp3a11*, *Cyp2b10*, *Cyp3a13*, and *Cyp2c55*. (B) Phase II conjugation genes *Ugt1a1* and *Ugt1a6*. (C) Hepatic transporter genes *Abcc2*, *Abcc3*, *Abcc4*, and *Oatp2*. Gene expression levels were quantified using normalized counts data determined by RNA-sequencing (n = 6-9 mice/group). Data are presented as Tukey box-and-whisker plots. Groups with the same letters are not significantly different, whereas groups with different letters differ significantly (*P* < 0.05), assessed using the Kruskal-Wallis H test with Conover post hoc comparisons. WT, wild type; KO, knockout; DKO, double knockout; *2c70*, *Cyp2c70*.
